# Antifungal Activity of Extracts, Fractions, and Constituents from *Coccoloba cowellii* Leaves

**DOI:** 10.3390/ph14090917

**Published:** 2021-09-10

**Authors:** Daniel Méndez, Julio C. Escalona-Arranz, Enrique Molina Pérez, Kenn Foubert, An Matheeussen, Emmy Tuenter, Ann Cuypers, Paul Cos, Luc Pieters

**Affiliations:** 1 Chemistry Department, Faculty of Applied Sciences, University of Camagüey, Carretera de Circunvalación Km 5½, Camagüey 74650, Cuba; daniel.mendez@reduc.edu.cu (D.M.); enrique.molina@reduc.edu.cu (E.M.P.); 2 Pharmacy Department, Faculty of Natural and Exact Sciences, Universidad de Oriente, Avenida Patricio Lumumba s/n, Santiago de Cuba 90500, Cuba; 3 Natural Products & Food Research and Analysis (NatuRA), Department of Pharmaceutical Sciences, University of Antwerp, Universiteitsplein 1, BE-2610 Antwerp, Belgium; kenn.foubert@uantwerpen.be (K.F.); emmy.tuenter@uantwerpen.be (E.T.); 4 Laboratory of Microbiology, Parasitology and Hygiene (LMPH), Faculty of Pharmaceutical, Biomedical and Veterinary Sciences, University of Antwerp, Universiteitsplein 1, BE-2610 Antwerp, Belgium; an.matheeussen@uantwerpen.be (A.M.); paul.cos@uantwerpen.be (P.C.); 5 Centre for Environmental Sciences, Campus Diepenbeek, Hasselt University, Agoralaan Building D, BE-3590 Diepenbeek, Belgium; ann.cuypers@uhasselt.be

**Keywords:** *Coccoloba cowellii*, Polygonaceae, UHPLC-ESI-QTOF-MS, methoxyflavonoids, antifungal

## Abstract

*Coccoloba cowellii* Britton (Polygonaceae, order Caryophyllales) is an endemic and critically endangered plant species that only grows in the municipality of Camagüey, a province of Cuba. A preliminary investigation of its total methanolic extract led to the discovery of promising antifungal activity. In this study, a bioassay-guided fractionation allowed the isolation of quercetin and four methoxyflavonoids: 3-*O*-methylquercetin, myricetin 3,3′,4′-trimethyl ether, 6-methoxymyricetin 3,4′-dimethyl ether, and 6-methoxymyricetin 3,3′,4′-trimethyl ether. The leaf extract, fractions, and compounds were tested against various fungi and showed strong in vitro antifungal activity against *Cryptococcus neoformans* and various *Candida* spp. with no cytotoxicity (CC_50_ > 64.0 µg/mL) on MRC-5 SV2 cells, determined by a resazurin assay. A *Candida albicans* SC5314 antibiofilm assay indicated that the antifungal activity of *C. cowellii* extracts and constituents is mainly targeted to planktonic cells. The total methanolic extract showed higher and broader activity compared with the fractions and mixture of compounds.

## 1. Introduction

Fungal infections represent a major health problem, with mortality rates comparable to those of tuberculosis or malaria, estimated at 1.5 million individuals per year [1]. The growing number of immunocompromised individuals due to the increase of organ transplantation, the prevalence of cancer and AIDS patients, and the ageing population have all contributed to this situation [2]. The predominant etiological agents of systemic fungal infections are species of *Candida*, *Aspergillus*, and *Cryptococcus*, representing over 90% of mycotic deaths [3]. *Candida* sp. stand out as the most common in the intensive care units, affecting individuals that undergo invasive clinical procedures and/or have experienced significant traumas requiring prolonged treatments [4]. *Candida albicans* is the most common (50–70%), producing more infections than all other *Candida* species combined [5]. *C. glabrata* is the second most dangerous species, with an increasing number of invasive candidiasis over the past several years [4].

Despite the negative impact that these fungi have on human health, currently, there are only three classes of antifungal agents available to treat serious *Candida* infections: azoles, echinocandins, and polyenes [6]. It is a worrying fact that almost all new approved drugs are based mainly on the old azole core structure [7]. In this context, natural plant derivatives constitute a promising resource available to the scientific community.

In a recent study, the total methanolic extract from the leaves of *Coccoloba cowellii* showed promissory antifungal activity against *C. albicans* and *Cryptococcus neoformans*, with IC_50_ values of 2.1 µg/mL and 4.1 µg/mL, respectively [8]. The major constituents of the aforementioned extract were glucuronides and glycosides of myricetin and quercetin; proanthocyanidins (tentatively characterized through HRMS); and epichatechin-3-*O*-gallate, catechin, epicatechin, and gallic acid (tentatively characterized using HRMS and authentic standards) [8]. The abundance of polyphenols in *C. cowellii* leaves and their recognised activity against fungi were the elements that allowed us to hypothesise that a bioassay-guided fractionation process could be a useful strategy to explore the potential of extracts, fractions, and constituents derived from this plant against a panel of *Candida* spp. which became the objective of the present work.

## 2. Results

### 2.1. Biofractionation Strategy

**Figure 1:**
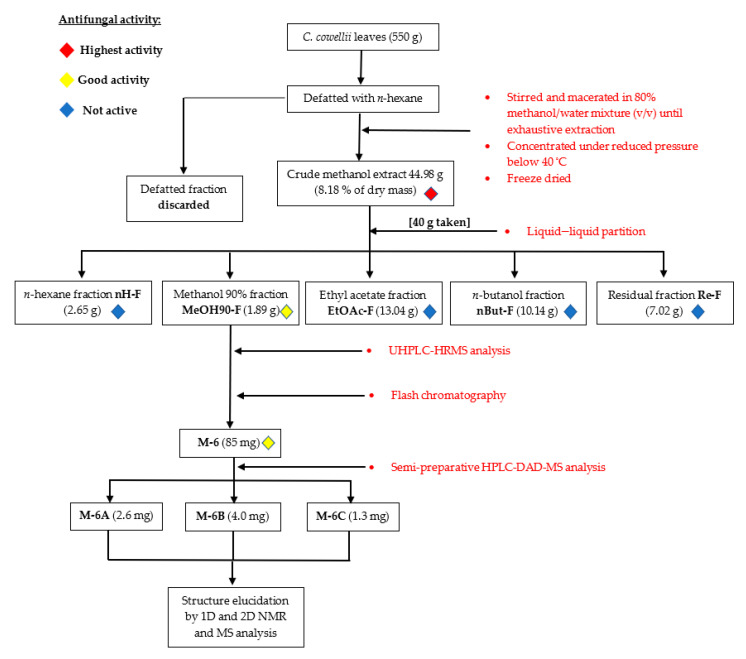
General scheme of the bioassay-guided fractionation performed on the methanolic extract from *C. cowellii* leaves.

The total methanolic extract from *C. cowellii* leaves (hereafter referred to as total extract) was subjected to bioassay-guided fractionation following the scheme shown in Figure 1. Given the promising results obtained for the total extract [8], the antifungal activity was employed as a guide for the fractionation and isolation of active compounds. Table 1 shows the results of the in vitro antifungal bioassays performed on the total extract and its fractions. Antifungal activity against a secondary panel of fungi (*Candida* spp. and *Aspergillus fumigatus*) was only determined when fractions were active against the first panel of fungi (*C. neoformans* and *C. albicans*). In general, samples were not active against *Aspergillus fumigatus* at concentrations of 64 µg/mL or lower. The total extract showed a strong antifungal effect against all strains with the exception of *C. tropicalis* and the previously mentioned *A. fumigatus*. Only the fraction MeOH90-F showed similar behaviour to the origin extract but with higher IC_50_ values.

At the same time, we intended to evaluate the selectivity of the antifungal activity using the cytotoxicity on human foetal lung fibroblasts (MRC-5 SV2 cells). None of the fractions showed cytotoxicity except for the *n*-hexane fraction (nH-F). Thus, the selectivity index of the total extract ranged from 160 (for *C. glabrata*) to 3 (for *C. tropicalis*), depending on the microorganism susceptibility. The indexes were consequently lowest for the MeOH90-F fraction, with values of 27 and 5 for *C. glabrata* and *C. parapsilosis*, respectively, while no activity was found against *C. tropicalis*. Nevertheless, in all cases, these values can be considered acceptable, evidencing a selective action on the microorganism tested.

Only the MeOH90-F fraction displayed significant antifungal activity, and it was thus chosen for further phytochemical characterization.

### 2.2. UHPLC-HRMS Characterization

**Figure 2:**
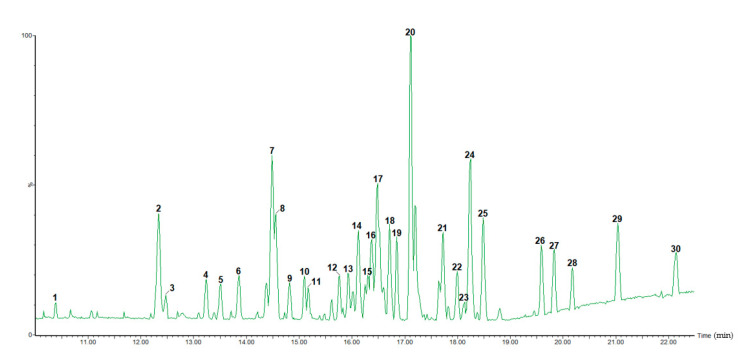
UHPLC-ESI-QTOF-MS base peak intensity (BPI) chromatogram (in negative ion mode) of the MeOH90-F fraction.

On the basis of the results obtained in the biological assays, the MeOH90-F fraction of *C. cowellii* was qualitatively analysed for its chemical composition using UHPLC-ESI-QTOF-MS in negative ionization mode. The base peak intensities (BPI, peaks 1 to 30 corresponding to Table 1) in negative ionization mode are shown in Figure 2.

The analysis and interpretation of the MS^E^ data allowed the identification of 21 phytochemical compounds from a total of 30 peaks (Table 2). The data from formerly identified phytochemicals in the *Coccoloba* genus and/or the Polygonaceae family were also utilised in the identification when applicable.

The fragment nomenclature for flavonoid glycosides was applied according to Vukics and Guttman [9]. The nomenclature used for lignin oligomers and fragments was taken from Morreel et al. [10]. The MS spectra are shown in the Supplementary Material (Figure S1a–o).

#### 2.2.1. Flavonoid Glycosides/Glucuronides

MS spectral data from peaks 1–3 matched with those previously reported for the total extract [8]. Peak 1 data fragmentation suggested (see Table 2) the presence of myricetin-*O*-glucuronide. The main fragment corresponded to the ion at *m*/*z* 317 [Y_0_]^−^ as a consequence of glucuronide loss, followed by a retro Diels-Alder (RDA) fragmentation generating the secondary fragments at *m*/*z* 287 [Y_0_-H-CO-H]^−^ and *m*/*z* 179 (^1,2^A^−^). On the other hand, the peak 2 and 3 data implied the presence of a quercetin-*O*-pentoside following similar fragmentation behaviour to compound **1**, but with the particularity that both homolytic and heterolytic loss of the sugar could be documented by the presence of ions at *m*/*z* 300 [Y_0_-H]^−·^ and *m*/*z* 301 [Y_0_]^−^, respectively. It was impossible to differentiate between these possible isomers; therefore, they were labelled as quercetin-*O*-pentoside 1 and 2, respectively.

#### 2.2.2. Lignin Oligomers

Peaks 4, 5, 7–11, and 19 all showed similar spectra and fragments. The fragmentation patterns matched the ones reported by Morreel et al. [10,11] for lignin oligomers. Table 3 shows the product ions of 4, 5, and 7–11, identified as trilignols. The fragment ion of *m*/*z* 195 (present in all the compounds) indicates that the 8-phenolic end (A^−^ fragment) corresponds to a G unit (guaiacyl, a unit derived from coniferyl alcohol) [12]. The fragmentation patterns (Figure S2a) were in correspondence with isomers of type G(8–O–4)X(8–5)X and G(8–O–4)X(8–8)X-containing trimers, with X being either an S unit (syringyl, a unit derived from sinapyl alcohol) or the previously mentioned G unit.

Peak 19 presented a [M-H]^−^ ion at *m*/*z* 809 and produced secondary fragmentations at *m*/*z* 761 [M-H-H_2_O-CH_2_O]^−^, *m*/*z* 613 (B^−^), *m*/*z* 565 (B^−^-H_2_O-CH_2_O), *m*/*z* 417 (second B^−^) and *m*/*z* 195 (A^−^). These fragments (Figure S2b) suggest a tetralignol type G(8–O–4)G(8–O–4)S(8–8)S structure.

#### 2.2.3. Methoxyflavonoids

Peaks 14, 15, 18, and 21–27 all showed one or several losses of 15 Da in their spectra, characteristic of methoxy compounds. The fragmentation patterns can be partially matched to the ones reported by Zhang et al. [13]. Table 4 shows the product ions of peaks 14, 15, 18, and 21–27, and the different losses were characteristic of polymethoxyflavonoids with at least three methoxy groups, except for peaks 14 and 18, which only contained one methoxy group.

Flavonoid glycosides and glucuronides with quercetin and myricetin aglycon moiety have already been reported in relatively high concentrations in the total extract of *C. cowellii* leaves [8] and in other species of the genus [14]. In fact, compounds **1**–**3** from this study matched the ones previously identified in the total extract [8]. The other compounds identified in the methanol 90% fraction were reported in this species for the first time.

Lignans and lignanoids are not commonly found in members of the Polygonaceae family and we could not find any report of this type of compound for the *Coccoloba* genus. Lignan glycosides have been isolated from the aerial parts of *Polygonum bellardii* [15]. Other lignin oligomers have been reported in *Polygonum perfoliatum* [16,17], *Polygonum aviculare* [18], *Rheum austral* [19], *Atraphaxis frutescens* [20], and *Polygonumcapitatum* [21]. On the other hand, methoxyflavonoids and their glycosides only occur in a few species of the Polygonaceae family, and we could not find any report of this type of compound in the *Coccoloba* genus. 5,8,3′,4′,5′-Pentahydroxy-3,7-dimethoxyflavone and 3-*O*-methylquercetin were isolated from an ethyl acetate extract and fractions of *Chorizanthe diffusa* [22]. Some methoxymyricetin derivatives, including 3-*O*-methylmyricetin-3′-*O*-β-d-xylopyranoside and 3-*O*-methylmyricetin, were isolated from the roots of *Pteroxygonum giraldii* [23]. Furthermore, the chemical investigation of the aerial parts of *Atraphaxis frutescens* resulted in the isolation of five 7-methoxyflavonols [20]. Isorhamnetin and 3,7-dihydroxy-5,6-dimethoxyflavone were among the compounds isolated from the dichloromethane extract of *Polygonum hydropiper* [24], and myricetin 3,7,3′,4′-tetramethyl ether has been isolated from *Polygonum viscosum* [25] and *Polygonum perfoliatum* [17].

The harsh growing conditions of *C. cowellii* can be associated with the presence of compounds unique to this species. This plant, strictly endemic to the savannas of north Camagüey, Cuba, only grows on serpentine soils and is subjected to almost constant drought and high levels of solar radiation (Figure S3). Its leaves are quite hard, with a lignified cuticula to prevent water loss. Therefore, these relatively rare compounds may be synthesised as a way to adapt to such severe adverse conditions [26].

These results portray a complex panorama: while the highly active total extract is rich in quercetin and myricetin glucosides/glucuronides and proanthocyanidins, its only active fraction is mainly comprised of lignans and methyl and methoxy derivatives of quercetin and myricetin. Only the aforementioned compounds **1**–**3** were common between both active tested extracts. In addition, and as can be seen in Figure 1, the lowest yield of extractable substances was obtained for the methanol 90% fraction, and therefore, active compounds must be present at very low concentrations in the crude extract. In fact, this could be an explanation for why such lignanoids and methoxyflavonoids were not detected during the study of the total extract. Despite these unfavourable conditions, biofractionation was pursued, aided by flash chromatography.

This second fractionation rendered 24 subfractions, which were all evaluated for their antifungal activity. Only one subfraction, M-6, showed some activity against *C. neoformans* and *C. glabrata* (Table 5). Therefore, this subfraction was selected for the isolation and characterization of its components through a semi-preparative HPLC-DAD-MS.

### 2.3. Isolated Compounds

**Figure 3:**
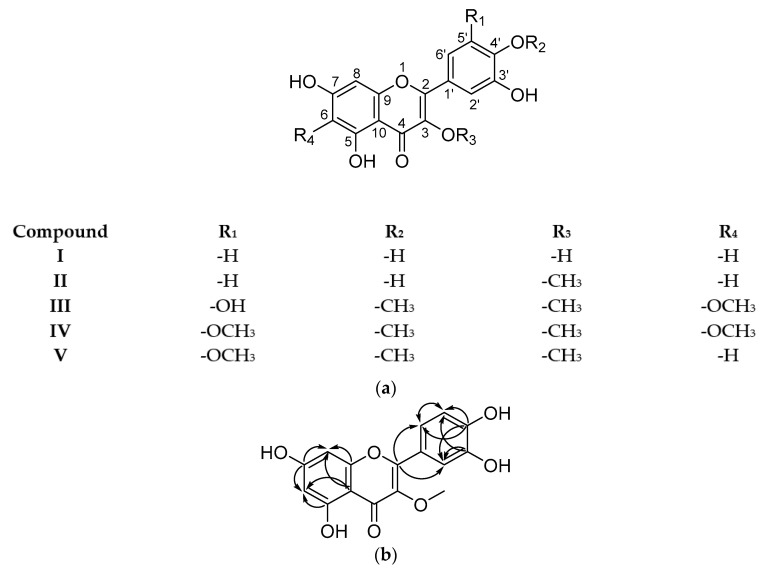
(**a**) Structures of compounds isolated from leaves of *C. cowellii*. (**b**) Correlations ^2^*J*_H-C_ and ^3^*J*_H-C_ observed in the HMBC spectra of compound **II**.

The semi-preparative HPLC-DAD-MS procedure rendered three main isolates (M-6A, M-6B, and M-6C) with yields of 2.6, 4.0, and 1.3 mg, respectively (Figure 1). The analysis of the ^1^H NMR spectra revealed that M-6A, M-6B, and M-6C were mixtures of two compounds in different ratios (Figure 3a). The low yields obtained did not allow further purification. Nevertheless, it was possible to determine the structures of both the major and minor compounds, except for the minor compound of M-6A. Employing the results obtained from SMART 2.1 (Tables S1–S5, Supplementary Information) and the molecular weight derived from the *m*/*z* value of the [M-H]^−^ peaks, a preliminary structure was drawn.

M-6A was identified as a mixture of quercetin (major, compound **I**) and an unidentified impurity (minor) (amorphous yellow powder, 2.6 mg). The NMR data (Figure S4) were similar to those previously reported in the literature for quercetin [27].

M-6B was identified as a mixture of 3-*O*-methylquercetin (major, compound **II**) and 6-methoxymyricetin 3,4′-dimethyl ether (minor, compound **III**) (amorphous yellow powder, 4.0 mg). The position of substituents was corroborated through ^2^*J*_H-C_ and ^3^*J*_H-C_ HMBC correlations (Figure 3b). NMR data (Figure S5) coincided with the data reported in the literature for 3-*O*-methylquercetin [28] and 6-methoxy-3-*O*-methylmearnsetin (6-methoxymyricetin 3,4′-dimethyl ether) [29]. Both compounds corresponded with peaks 14 and 15 proposed in the UHPLC-HRMS analysis.

M-6C was identified as a mixture of 6-methoxymyricetin 3,3′,4′-trimethyl ether (major, compound **IV**), and myricetin 3,3′,4′-trimethyl ether (minor, compound **V**) (amorphous yellow powder, 1.3 mg). The NMR spectral data (Figure S6) were in agreement with the assignments reported in the literature for 5,7,3′-trihydroxy-3,6,4′,5′-tetramethoxyflavone (6-methoxymyricetin 3,3′,4′-trimethyl ether) [25] and for myricetin 3,3′,4′-trimethyl ether [30]. Both compounds corresponded with peaks 21 and 22 proposed in the UHPLC-HRMS analysis.

For the structure elucidation, NMR data of compounds with a similar chemical backbone were consulted for comparison [31,32,33].

Table 5 shows the activities of subfraction M-6 and the mixtures of compounds defined as M-6A, M-6B, and M-6C. As can be seen, the antifungal activity of the binary mixture M-6C against *C. glabrata* and *C. neoformans* was approximately the same as the activity of the methanol 90% fraction, but the activity remained lower than that of the total extract. Furthermore, it was noted that the M-6 fraction and the three mixtures of compounds were active against *C. glabrata* specifically. The increasing resistance of this *Candida* species against azole compounds [4] and echinocandins [34] necessitate the search for novel compounds that can be used to treat infections caused by this fungus.

Methoxyflavonoids (specifically, derivatives of the flavonols quercetin and myricetin) seem to be responsible for the observed activity. Antifungal activity has previously been reported for these types of compounds [35,36]. The analysis of *Limonium caspium* (Plumbaginaceae) showed that the compound 5-methylmyricetin exhibited good antifungal activity against *C. glabrata*, with an IC_50_ value of 6.79 μg/mL [37]. From *Combretum zeyheri* (Combretaceae), the compound 5-hydroxy-7,4′-dimethoxyflavone was found to be active against *C. albicans* using the broth dilution method. These substances showed synergistic activity when combined with miconazole, completely inhibiting *C. albicans* growth after only 4 h of incubation [38]. The study of the plant *Kaempferia parviflora* (Zingiberaceae) allowed the identification of 3,5,7,4′-tetramethoxyflavone and 5,7,4′-trimethoxyflavone as acceptable antifungal agents against *C. albicans*, with respective IC_50_ values of 39.71 and 17.63 μg/mL [39].

### 2.4. Antibiofilm Screening Assay

Plant extracts and/or their isolated compounds can act as antimicrobials via different mechanisms. Biofilm disruption is one of the most explored, and the active extracts/fractions/compounds were tested for this mode of action. The biofilm screening assay was realised by employing the *C. albicans* SC5314 strain [40]. The formation of fungal biofilms decreased overall susceptibility from both host defences and antimicrobial therapies [41]. Natural products have been reported to demonstrate antibiofilm activity, which is relevant because developing resistance to these kinds of molecules is rare [42]. The results showed (Table 6) that only the total extract showed low activity against the *C. albicans* biofilms, while the rest of the samples showed no effect at the tested concentrations (0.25 to 64 µg/mL to all the samples except for M-6C due to the low amount).

This assay indicated that the antifungal activity of the total extract of *C. cowellii* on *Candida* species is mainly targeted to planktonic cells and has rather low activity against biofilm colonies, at least in the conditions established in these experiments. The mechanism(s) of action of the total extract and active fractions could thus be related to inducing the death of free-living cells and not the disruption of cell-to-cell communication and biofilm association.

## 3. Discussion

The bioassay-guided fractionation performed on *C. cowellii* leaves led to the isolation and tentative identification of at least 21 new compounds in this species; nevertheless, the isolation of the compounds responsible for high antifungal activity shown by the total extract was not successful. Unfortunately, this is not an uncommon situation. Bioassay-guided fractionation of plant extracts is not always effective. This procedure can lead to failures in the isolation of active compounds and losses of activity [43]. The probable degradation of the compounds during the purification process, the difficulty related to isolating bioactive compounds present in low concentrations, and/or the loss of other substances responsible for potential synergistic effects are some of the causes referred to in the literature [44]. In any case, the higher and broader activity of the total extract of *C. cowellii* compared with the fractions and mixtures of compounds can be associated with any of these aforementioned events.

According to the previous analysis, flavonoid glycosides or glucuronides as well as proanthocyanidins are the main compounds of the total extract *of C. cowellii* leaves [8]. These compounds have a broad spectrum of biological activities, including antifungal activity [45,46]. This mixture of different kinds of polyphenols can contribute to the overall antifungal activity, considering that the different groups can have different underlying mechanisms of action [35]. Proanthocyanidins have shown synergic effects with various commercial antifungal agents. Catechin and epigallocatechin gallate have shown synergism with fluconazole. These compounds induce the activation of the phospholipid phosphatidylserine, which inhibits fatty acid synthase [47], supporting in this way the action mechanism of fluconazole. In a preclinical study of disseminated candidiasis, epigallocatechin-*O*-gallate administered with amphotericin B showed a synergistic interaction against *C. albicans*. The results of the assay showed that epigallocatechin-*O*-gallate exclusively inhibits the hyphal formation and ergosterol synthesis of the fungi [48]. The synergic effect of these main compounds, together with the effect of the methoxyflavonoids (see Table 3), is a plausible explanation for the high antifungal activity identified in the total extract of *C. cowellii* leaves. In any case, the extract is a promising candidate for the treatment of fungal diseases, either alone or mixed with commercial antifungals as a way to increase their effectiveness and/or decrease the required doses. Confirmatory assays will be necessary to corroborate these hypotheses.

## 4. Materials and Methods

### 4.1. Chemicals and Plant Material

Acros Organics (Geel, Belgium) and Fisher Scientific (Leicestershire, UK) were the companies from which all analytical grade solvents, such as *n*-hexane, chloroform, dichloromethane, ethyl acetate, isopropyl alcohol, *n*-butanol, methanol, and dimethyl sulfoxide (DMSO), were purchased. HPLC solvents, such as methanol and acetonitrile, were purchased from Fisher Scientific, while UPLC-grade solvents, such as acetonitrile and formic acid, were purchased from Biosolve (Valkenswaard, the Netherlands). Finally, methanol-*d*4 (≥99.8% D) was obtained from Sigma-Aldrich (St. Louis, MO, USA); Milli-Q quality water was dispensed using a Milli-Q system from Millipore (Bedford, MA, USA) and subsequent membrane filtration at 0.22 μm was performed before use. *Coccoloba cowellii* leaves were collected near Albaisa in the municipality of Camagüey, Cuba (Lat. 21.43615, Long. −77.83253), and were taxonomically identified by the curator of the herbarium “Julián AcuñaGalé” of the University of Camagüey (HIPC, http://sweetgum.nybg.org/science/ih/herbarium-details/?irn=124935 (accessed on 20 August 2021)), where a sample specimen (number 12057) was deposited.

### 4.2. Leaf Extraction and Bioassay-Guided Fractionation

The plant material was processed, and the total extract was obtained as previously described [8]. The total extract (40.00 g) was dissolved in acidic (pH < 3) methanol 50% and partitioned with dichloromethane. The dichloromethane residue was concentrated and partitioned between *n*-hexane and methanol 90%. Next, the extract was basified with concentrated ammonia until it reached a pH > 9, and partitioning with ethyl acetate and then *n*-butanol was performed. The yield of all the fractions was 2.65 g for the *n*-hexane fraction (nH-F), 1.89 g for the methanol 90% fraction (MeOH90-F), 13.04 g for the ethyl acetate fraction (EtOAc-F), 10.14 g for the *n*-butanol fraction (nBut-F), and 7.02 g for the residual fraction (Re-F). The total yield of the fractionation was 86.9%. nH-F, MeOH90-F, EtOAc-F, and nBut-F were concentrated under reduced pressure at 40 °C and then stored at −20 °C until further use.

### 4.3. Antifungal Assay

The microdilution method with resazurin (redox indicator) in sterile 96-well microplates was the assay used to determine antifungal activity. This was performed according to the protocols of the Laboratory of Microbiology, Parasitology, and Hygiene (LMPH) as previously reported [8,49]. Miconazole was used as a positive control.

#### Microorganisms and Dilutions

The microorganisms used in the study were obtained from the culture collection of the Laboratory of Microbiology, Parasitology, and Hygiene (LMPH of the University of Antwerp). The strains of *Candida albicans* ATCC B59630 (azole-resistant), *Candida glabrata* ATCC B63155, *Candida parapsilosis* ATCC B66126, *Candida tropicalis* ATCC CDC44 as well as *Aspergillus fumigatus* ATCC B42928 and *Cryptococcus neoformans* ATCC B66663 were used for in vitro screening of antifungal activity. The LMPH protocols established for the culture of the microorganisms and the dilutions of the samples were followed, as performed and reported in previous publications [8,49]. Tested sample concentrations ranged from 0.25 to 128 μg/mL.

### 4.4. Antibiofilm Screening Assay

The antibiofilm assay was performed following the LMPH protocols previously reported [50]. A *Candida albicans* SC5314 overnight culture, grown in RPMI, was diluted to an optical density (OD) of 0.04–0.05 in RPMI medium, and 95 μL of this suspension was added to a 96-well plate. Then, 5 μL samples and control (miconazole) were added (in a final concentration range from 0.25 to 128 μg/mL). The plate was wrapped in parafilm and placed in a styropor box, a cup of MilliQ water was added, and the box was placed in a shaking incubator at 37 °C and 25 rpm. After 24 h of incubation, the medium was carefully removed with a vacuum pump, avoiding contact of pipette tips with the biofilms. Finally, the biofilms were washed and quantified by adding 150 μL of resazurin solution (1/10 in PBS) to each well. The plate was wrapped in aluminium foil and incubated in the dark at 37 °C for 1 h. The fluorescence was measured with a microplate reader (TECAN GENios, Männedorf, Switzerland) at a λ_ex_ of 550 nm and a λ_em_ of 590 nm.

### 4.5. Cytotoxicity Assay

Human foetal lung fibroblasts (MRC-5 SV2 cells) were purchased from the ATCC (American Type Culture Collection). For their culture and assay, the protocols of the Laboratory of Microbiology, Parasitology, and Hygiene (LMPH) were followed, as reported in previous publications [8,49]. The 50% cytotoxic concentration (CC_50_), resulting from the % reduction of cell growth/viability compared to control wells, is reported. Tamoxifen was used as a reference drug.

### 4.6. UHPLC-HRMS Characterization

The UHPLC-ESI-QTOF-MS analysis of *C. cowellii* extract was carried out as previously reported [8]. H_2_O + 0.1% FA (A) and ACN + 0.1% FA (B) were used as mobile phase, and a flow gradient of (min/B%): 0.0/2.0, 1.0/2.0, 14.0/26.0, 24.0/65.0, 26.0/100.0, 29.0/100.0, 31.0/2.0, and 36.0/2.0 was used. MS^E^ in negative ionization mode (two analyses per mode), was recorded. A collision energy ramp from 10 to 30 V was applied to obtain additional structural information. Leucine-encephalin was used as a blocking mass. UV detection was performed at a wavelength equivalent to 360 nm.

#### Data Processing

The UHPLC-ESI-QTOF-MS raw data were analysed using MassLynx 4.1 Copyright © 2014 Waters Inc. (Waters, Milford, MA, USA). For identification, the following public databases were consulted: PubChem (https://pubchem.ncbi.nlm.nih.gov/ (accessed on 20 August 2021)), ChemSpider (https://www.chemspider.com/ (accessed on 20 August 2021)), MassBank of North America (MoNA) (http://mona.fiehnlab.ucdavis.edu/ (accessed on 20 August 2021)), and NIST Mass Spectrometry Data Center (http://chemdata.nist.gov/ (accessed on 20 August 2021)).

### 4.7. Isolation of Constituents from Active Fractions

In the fractionation of the MeOH90-F fraction (1.7 g), selected as the most active, flash chromatography was applied on a GraceResolv 80 g silica column using a RevelerisiES system (Columbia, MD, USA). A gradient program consisting of dichloromethane (A), ethyl acetate (B), and methanol (C) as mobile phases and a flow rate of40 mL/min were used (Figure S7). These solvents were applied as follows: 0 to 6 min 100% A, 0% B, and 0% C; 6 to 40 min linear changing until 0% A and 100% B; 40 to 45 min 0% A, 100% B, and 0% C; 45 to 81 min linear changing until 0% B and 100% C. An evaporative light scattering detector (ELSD) and UV absorption at 254 and 366 nm were used as the detection methods. According to their TLC profile, all collected subfractions were pooled for a total of 24 subfractions (M-1 to M-24).

On the basis of its chromatographic profile, fraction size, and biological activity, subfraction M-6 (85 mg) was selected for further purification by semi-preparative HPLC-DAD-MS (Waters, Milford, MA, USA). A C18 Luna (250 mm × 10.0 mm, particle size 5 μm) from Phenomenex (Utrecht, The Netherlands) was used as a column. As in previous experiments, H_2_O/0.1% formic acid (A) and acetonitrile (B) were used as mobile phases; consistent linear gradients were applied as follows: 0–5 min 25% B, 38 min 45% B, 40–45 min 95% B, 50–60 min 25% B, and the flow rate was 3.0 mL/min.

Mass spectra in negative ESI mode, MS scan range: *m*/*z* 150 to 750; V_capillary_ 3.00 kV, V_cone_ 50 V, V_extractor_ 3 V, VRF Lens 0.2 V, T_source_ 135 °C, T_desolvation_ 400 °C, desolvation gas flow 750 L/h, and cone gas flow 0 L/h were obtained, along with the DAD spectrum, which was recorded in the range between 200 and 450 nm. The *m*/*z* value and the UV spectrum conditioned the selection of the peaks of interest. In turn, *m*/*z* values that exceeded the set threshold served as a trigger for eluate collection. Three compounds were provisionally isolated: M-6A (2.6 mg), M-6B (4.0 mg), and M-6C (1.3 mg).

### 4.8. Structure Elucidation

A Bruker DRX-400 instrument (Rheinstetten, Germany), equipped with either a 3 mm broadband inverse (BBI) probe or a 5 mm dual ^1^H/^13^C probe was used to record the NMR spectra, using standard Bruker pulse sequences. 1D ^1^H (400 MHz) and ^13^C (100 MHz), as well as DEPT-135, DEPT-90, and 2D NMR (COSY, HSQC, and HMBC) experiments, were used to characterize the isolated compounds. Bruker TopSpin® software version 4.0.8 for Windows (Billerica, MA, USA) was selected for NMR data processing. In turn, the prediction of chemical structures from NMR data was performed using the NMR-based machine learning tool “Small Molecule Accurate Recognition Technology” (SMART 2.1, available at https://smart.ucsd.edu/classic (accessed on 20 August 2021)). These proposed results were contrasted with those obtained from mass spectra derived from the semi-preparative HPLC-DAD-MS system.

Quercetin (compound I, yellow powder (2.6 mg)): ^1^H NMR (400 MHz, MeOD): δ 6.20 (s, 1H, H-6); 6.40 (s, 1H, H-8); 6.90 (d, *J =* 8.4 Hz, 1H, H-5′); 7.65 (dd, *J =* 1.7, 8.4 Hz, 1H, H-6′); 7.75 4.86 (d, *J =* 1.7 Hz, 1H, H-2′). ^13^C NMR (100 MHz, MeOD): δ 93.8 (C-8), 98.8 (C-6), 104.0 (C-10), 115.7 (C-2′), 115.8 (C-5′), 121.3 (C-6′), 123.7 (C-1′), 145.8 (C-3′), 147.5 (C-2), 148.3 (C-4′), 157.8 (C-9), 162.0 (C-5), 165.1 (C-7). ESI-MS (negative mode): *m*/*z* 301 [M-H]^−^ (C_15_H_9_O_7_).

3-*O*-Methylquercetin (compound II, yellow powder (4.0 mg)): ^1^H NMR (400 MHz, MeOD): δ 3.79 (s, 3H, 3-OCH_3_); 6.22 (s, 1H, H-6); 6.41 (s, 1H, H-8); 6.92 (d, *J =* 8.5 Hz, 1H, H-5′); 7.54 (dd, *J =* 1.8, 8.5 Hz, 1H, H-6′); 7.64 (d, *J =* 1.8 Hz, 1H, H-2′). ^13^C NMR (100 MHz, MeOD): δ 60.5 (OCH_3_), 94.7 (C-8), 99.8 (C-6), 105.9 (C-10), 116.5 (C-2′), 116.5 (C-5′), 122.3 (C-6′), 123.0 (C-1′), 139.7 (C-3), 146.5 (C-3′), 150.2 (C-2), 150.0 (C-4′), 158.5 (C-9), 163.1 (C-5), 166.0 (C-7). ESI-MS (negative mode): *m*/*z* 315 [M-H]^−^ (C_16_H_11_O_7_).

6-Methoxymyricetin 3,4′-dimethyl ether (compound III, yellow powder (4.0 mg)): ^1^H NMR (400 MHz, MeOD): δ 3.81(s, 3H, 3-OCH_3_); 3.89 (s, 3H, 6-OCH_3_); 3.90 (s, 3H, 4′-OCH_3_); 6.52 (s, 1H, H-8); 7.19 (s, 1H, H-2′); 7.19 (s, 1H, H-6′). ^13^C NMR (100 MHz, MeOD): δ 60.7 (OCH_3_), 60.8 (OCH_3_), 61.0 (OCH_3_), 95.0 (C-8), 106.4 (C-10), 109.2 (C-2′), 109.2 (C-6′), 127.0 (C-1′), 132.7 (C-6), 139.6 (C-4′), 140.1 (C-3), 152.0 (C-3′), 152.0 (C-5′), 153.9 (C-9), 157.5 (C-2), 158.9 (C-7), 180.5 (C-4). ESI-MS (negative mode): *m*/*z* 375 [M-H]^−^ (C_18_H_15_O_9_).

6-Methoxymyricetin 3,3′,4′-trimethyl ether (compound IV, yellow powder (1.3 mg)): ^1^H NMR (400 MHz, MeOD): δ 3.83 (s, 3H, 3-OCH_3_); 3.89 (s, 3H, 4′-OCH_3_); 3.89 (s, 3H, 6-OCH_3_); 3.93 (s, 3H, 5′-OCH_3_); 6.54 (s, 1H, H-8); 7.30 (s, 1H, H-2′); 7.30 (s, 1H, H-6′). ^13^C NMR (100 MHz, MeOD): δ 55.2 (OCH_3_), 59.4 (OCH_3_), 59.6 (OCH_3_), 59.6 (OCH_3_), 93.7 (C-8), 103.9 (C-6′), 105.1 (C-10), 109.6 (C-2′), 125.5 (C-1′), 131.1 (C-6), 138.6 (C-3), 139.0 (C-4′), 150.5 (C-3′), 152.7 (C-9), 153.2 (C-5′), 155.8 (C-2), 157.9 (C-7), 179.0 (C-4). ESI-MS (negative mode): *m*/*z* 389 [M-H]^−^ (C_19_H_17_O_9_).

Myricetin 3,3′,4′-trimethyl ether (compound V, yellow powder (1.3 mg)): ^1^H NMR (400 MHz, MeOD): δ 3.83 (s, 3H, 3-OCH_3_); 3.89 (s, 3H, 4′-OCH_3_); 3.93 (s, 3H, 5′-OCH_3_); 6.23 (s, 1H, H-6); 6.43 (s, 1H, H-8); 7.30 (d, *J =* 2.1 Hz, 1H, H-2′); 7.30 (d, *J =* 2.1 Hz, 1H, H-6′). ^13^C NMR (100 MHz, MeOD): δ 55.2 (OCH_3_), 59.4 (OCH_3_), 59.6 (OCH_3_), 93.5 (C-8), 98.6 (C-6), 103.9 (C-6′), 109.6 (C-2′), 125.8 (C-1′), 138.6 (C-3), 139.0 (C-4′), 150.5 (C-3′), 153.2 (C-5′), 155.8 (C-2). ESI-MS (negative mode): *m*/*z* 359 [M-H]^−^ (C_18_H_15_O_8_).

### 4.9. Statistical Analysis

GraphPad Prism V8 Software for Windows (GraphPad, San Diego, CA, USA) was employed for all statistical analyses. The results were analysed and expressed as the means ± standard deviation (SD) of three different replicates.

## 5. Conclusions

In this study, five secondary metabolites were isolated and characterized from the MeOH90-F fraction of the total extract of *C. cowellii* by means of a combined methodology of NMR and MS analysis. All five are reported here for the first time for both the plant and the genus. Another 16 compounds were tentatively characterized employing UHPLC-HRMS. *C. cowellii* extract was confirmed to have good antifungal activity against a second fungal/yeast panel, while fractions and mixtures of compounds obtained from the bioassay-guided fractionation showed acceptable activity specifically against *C. glabrata* and C. *neoformans.* These results highlight the possible use of this plant as a natural antifungal and contribute to a better understanding of the phytochemistry and biological activities of the genus *Coccoloba.* At the same time, they suggest the probable synergistic effect that the combination of different types of polyphenols may show in inhibiting fungal growth.

## Figures and Tables

**Table 1:** In vitro antifungal and cytotoxic activity of the total extract and fractions from *C. cowellii* leaves.

Test Sample	Cytotoxicity (CC_50_ µg/mL)	Antifungal Screening (IC_50_ µg/mL)					
	MRC-5	*Aspergillus fumigatus*	*Cryptococcus neoformans*	*Candida albicans*	*Candida parapsilosis*	*Candida glabrata*	*Candida tropicalis*
TE	>64.0	>64.0	2.7 ± 2.0	1.7 ± 0.6	8.5 ± 0.5	0.4 ± 0.0	21.2 ± 1.8
MeOH90-F	>64.0	>64.0	10.5 ± 1.0	8.3 ± 0.9	13.3 ± 1.1	2.4 ± 0.4	>64.0
nH-F	29.3 ± 1.5	Nd	>64.0	>64.0	Nd	Nd	Nd
EtOAc-F	>64.0	Nd	>64.0	>64.0	Nd	Nd	Nd
nBut-F	>64.0	Nd	>64.0	>64.0	Nd	Nd	Nd
Re-F	>64.0	Nd	>64.0	>64.0	Nd	Nd	Nd
Miconazole	19.8 ± 0.7	3.7 ± 0.5	0.2 ± 0.0	3.4 ± 0.2	1.1 ± 0.1	0.2 ± 0.0	3.6 ± 1.0

TE: total extract; MeOH90-F: methanol 90% fraction; nH-F: *n*-hexane fraction; EtOAc-F: ethyl acetate fraction; nBut-F: *n*-butanol fraction; Re-F: residual fraction; MRC-5: human fetal lung fibroblasts; Nd: not determined. Values are means ± SD of three replicates.

**Table 2:** Chemical composition of the methanol 90% fraction of the total extract of *C. cowellii*.

Peak No.	Rt (min)	[M-H]^−^ (*m*/*z*)	Theoretical Mass (*m*/*z*)	Accuracy (ppm)	MS/MS Ions	MF	Tentative Identification
1	10.39	493.0620	493.0618	0.4	317.0281/315.0105/287.0563/178.9872	C_21_H_17_O_14_	Myricetin-*O*-glucuronide
2	12.33	433.0775	433.0771	0.9	301.0344/300.0273/271.0247/255.0294	C_20_H_17_O_11_	Quercetin-*O*-pentoside 1
3	12.47	433.0763	433.0771	−1.8	301.0357/300.0253/271.0255/255.0187	C_20_H_17_O_11_	Quercetin-*O*-pentoside 2
4	13.23	555.2225	555.2230	−0.9	507.2011/477.1888	C_30_H_35_O_10_	Trilignol G(8–O–4)G(8–5)G
5	13.50	555.2216	555.2230	−2.5	507.1984/477.1816/341.1288/329.1320/195.0650/165.0273	C_30_H_35_O_10_	Trilignol G(8–O–4)G(8–5)G
6	13.85	312.1228	312.1236	−2.6	197.8091/195.8118/116.9287	-	Unknown
7	14.49	585.2429	585.2430	−0.2	537.2122/507.1984/371.1458/359.1454/195.0658/165.0374	C_31_H_37_O_11_	Trilignol G(8–O–4)X(8–8)X
8	14.56	583.2163	583.2179	−2.7	535.1965/505.1852/369.1333/357.1330/195.0658/165.0301	C_31_H_35_O_11_	Trilignol G(8–O–4)S(8–5)G
9	14.82	585.2329	585.2336	−1.2	537.2112/507.1821/359.1410/195.0639/165.0157	C_31_H_37_O_11_	Trilignol G(8–O–4)X(8–8)X
10	15.09	585.2331	585.2336	−0.9	537.2020/507.1826/371.1437/359.1445/195.0655/165.0552	C_31_H_37_O_11_	Trilignol G(8–O–4)X(8–8)X
11	15.17	583.2172	583.2179	−1.2	369.1325/357.1325/195.0656/165.0551	C_31_H_35_O_11_	Trilignol G(8–O–4)S(8–5)G
12	15.76	583.2177	583.2179	−0.3	565.2036/489.1883/477.1877/417.1481/371.1414/359.1383/193.0497	-	Unknown
13	16.02	583.2177	583.2179	−0.3	581.1965/535.1947/387.1389/367.1148/195.0648/165.0052	-	Unknown
14	16.12	315.0513	315.0505	2.5	300.0270/271.0238	C_16_H_11_O_7_	3-*O*-Methylquercetin
15	16.24	375.0704	375.0716	−3.2	360.0495/345.0239/330.0117/327.1691/317.0265/300.0250/171.0929	C_18_H_15_O_9_	6-Methoxymyricetin 3,4′-dimethyl ether
16	16.37	327.2177	327.2171	1.8	285.0412/256.0378/229.1443/211.1334/171.1033	-	Unknown
17	16.49	345.0612	345.0610	0.6	301.0422	-	Unknown
18	16.71	315.0510	315.0505	1.6	300.0278/271.0252/255.0304/243.0296	C_16_H_11_O_7_	*O*-Methylquercetin
19	16.85	809.3019	809.3021	−0.2	761.2747/613.2260/565.2047/417.1499/195.0660	C_42_H_49_O_16_	Tetralignol G(8–O–4)G(8–O–4)S(8–8)S
20	17.11	331.2645	331.2637	2.4	313.2187	-	Unknown
21	17.73	389.0888	389.0873	3.9	374.0632/359.0416/331.0509/316.0201/287.2135	C_19_H_17_O_9_	6-Methoxymyricetin 3,3′,4′-trimethyl ether
22	17.99	359.0764	359.0767	−0.8	344.0509/329.0413/301.0361/286.0089/273.0367/257.9776/242.0100/222.9688/162.8474	C_18_H_15_O_8_	Myricetin 3,3′,4′-trimethyl ether
23	18.13	359.0751	359.0767	−4.5	344.0493/329.0565/301.0364/286.0081/257.9645/222.9675/162.8543	C_18_H_15_O_8_	Methoxyquercetin dimethyl ether 1
24	18.25	389.0870	389.0873	−0.8	374.0634/359.0398/344.0168/316.0218/300.9995/245.0086	C_19_H_17_O_9_	Methoxymyricetin trimethyl ether
25	18.48	359.0771	359.0767	1.1	344.0535/329.0302/301.0346/286.0122/258.0163	C_18_H_15_O_8_	Methoxyquercetindimethyl ether 2
26	19.58	403.1047	403.1029	4.5	388.0773/373.0557/358.0301/345.0566/330.0363/315.0175/257.9344/222.9669	C_20_H_19_O_9_	Methoxymyricetin tetramethyl ether
27	19.83	373.0939	373.0923	4.3	358.0623/343.0453/328.0199/315.0660/300.0232/285.0035/257.9385/222.9662	C_19_H_17_O_8_	Myricetin tetramethyl ether
28	20.18	349.2156	349.2168	−3.4	313.2335/251.1598/199.8060/197.8089/195.8118/116.9286	-	Unknown
29	21.03	721.3658	721.3647	1.5	675.3555/415.1435/397.1342/277.1996/257.9326/222.9646	-	Unknown
30	22.15	559.3133	559.3118	2.7	567.2234/505.1054/320.0494/277.2092/257.9327/222.9659	-	Unknown

Rt: retention time; MF: molecular formula.

**Table 3:** MS/MS fragmentation patterns of trilignols of the methanol 90% fraction of the total extract of *C. cowellii*.

Ions	Peak 4	Peak 5	Peak 7	Peak 8	Peak 9	Peak 10	Peak 11
[M-H]^−^	555 (18)	555 (49)	585 (41)	583 (38)	585 (36)	585 (58)	583 (45)
[M-H-H_2_O]^−^	537 (4)	537 (12)	567 (2)	565 (3)	567 (2)	567 (4)	565 (7)
[M-H-H_2_O-CH_2_O]^−^	507 (100)	507 (90)	537 (100)	535 (100)	537 (100)	537 (18)	535 (15)
[M-H-H_2_O-CH_2_O-CH_2_O]^−^	477 (27)	477 (55)	507 (23)	505 (20)	507 (13)	507 (12)	505 (10)
A^−^	195 (13)	195 (100)	195 (27)	195 (37)	195 (32)	195 (100)	195 (100)
A^−^-CH_2_O	165 (13)	165 (78)	165 (19)	165 (31)	165 (22)	165 (67)	165 (70)
B^−^-H_2_O	341 (7)	341 (46)	371 (24)	369 (41)	371 (3)	371 (66)	369 (90)
B^−^-CH_2_O	329 (10)	329 (77)	359 (30)	357 (39)	359 (50)	359 (82)	357 (83)

The relative intensity of the product ions compared with the base peak is given in parentheses.

**Table 4:** MS/MS fragmentation patterns of methoxyflavonoids of the methanol 90% fraction of the total extract of *C. cowellii*.

Ions	Peak 14	Peak 15	Peak 18	Peak 21	Peak 22	Peak 23	Peak 24	Peak 25	Peak 26	Peak 27
[M-H]^−^	315 (15)	375 (12)	315 (25)	389 (19)	359 (39)	359 (19)	389 (12)	359 (12)	403 (19)	373 (11)
[M-H-CH_3_.]^−^	300 (100)	360 (36)	300 (66)	374 (44)	344 (65)	344 (40)	374 (22)	344 (7)	388 (54)	358 (11)
[M-H-2CH_3_.]^−^	-	345 (100)	-	359 (100)	329 (100)	329 (100)	359 (100)	329 (100)	373 (100)	343 (100)
[M-H-3CH_3_.]^−^	-	330 (17)	-	344 (13)	314 (15)	314 (16)	344 (36)	314 (7)	358 (34)	328 (12)
[M-H-2CH_3_.-CO]^−^	-	317 (15)	-	331 (38)	301 (54)	301 (49)	331 (7)	301 (16)	345 (33)	315 (10)
[M-H-2CH_3_.-CO-CH_3_.]^−^	-	-	-	316 (32)	286 (42)	286 (43)	316 (44)	286 (19)	330 (69)	300 (13)
[M-H-2CH_3_.-CO-2CH_3_.]^−^	-	-	-	301 (17)	-	-	301 (16)	-	315 (34)	285 (7)
[M-H-2CH_3_.-H_2_O]^−^	-	327 (24)	-	341 (17)	-	-	-	-	-	-
[M-H-CH_3_.-HCO.]^−^	271 (12)	-	271 (100)	-	-	-	-	-	-	-
Others		300 (22) 171 (24)	255 (42) 243 (20)	287 (62) 245 (15)	273 (46) 258 (62)	258 (48) 223 (22) 163 (18)	245 (12)	258 (14)	258 (37)	

The relative intensity of the product ions compared with the base peak is given in parentheses.

**Table 5:** In vitro antifungal and cytotoxic activity of subfraction M-6 and the binary mixtures M-6A, M-6B, and M-6C from *C. cowellii* leaves.

Test Sample	Cytotoxicity (CC_50_ µg/mL)	Antifungal Screening (IC_50_ µg/mL)					
	MRC-5	*A. fumigatus*	*C. neoformans*	*C. albicans*	*C. parapsilosis*	*C. glabrata*	*C. tropicalis*
M-6	>64.0	>64.0	50.3 ± 9.2	>64.0	>64.0	9.5 ± 1.1	>64.0
M-6A	>64.0	>64.0	>64.0	>64.0	>64.0	7.9 ± 1.3	>64.0
M-6B	>64.0	>64.0	59.5 ± 6.4	>64.0	>64.0	9.1 ± 1.8	>64.0
M-6C	>32.0	>32.0	8.3 ± 0.0	>32.0	>32.0	3.8 ± 0.0	>32.0
Miconazole	19.8 ± 0.7	3.7 ± 0.5	0.2 ± 0.0	3.4 ± 0.2	1.1 ± 0.1	0.2 ± 0.0	3.6 ± 1.0

M-6: subfraction of the flash chromatography of MeOH90-F; M-6A: compound **I** and minor impurity; M-6B: mixture of compounds **II** and **III**; M-6C: mixture of compounds **IV** and **V**. MRC-5: human fetal lung fibroblasts. Values are means ± SD of three replicates.

**Table 6:** In vitro antibiofilm activity of the total extract and fractions from *C. cowellii* leaves.

Test Sample	Antibiofilm Screening (IC_50_ µg/mL)
	*Candida albicans* SC5314
TE	49.73 ± 2.1
MeOH90-F	>64.00
M-6	>64.00
M-6A	>64.00
M-6B	>64.00
M-6C	>32.00
Miconazole	0.60 ± 0.6

TE: total extract; MeOH90-F: methanol 90% fraction of TE: M-6: subfraction of the flash chromatography of MeOH90-F; M-6A: compound **I** and minor impurity; M-6B: mixture of compounds **II** and **III**; M-6C: mixture of compounds **IV** and **V**. Values are means ± SD of three replicates.

## Data Availability

The data presented in this study are available in Supplementary Materials.

## Supplementary Materials

The following are available online at https://www.mdpi.com/article/10.3390/ph14090917/s1, Figure S1: Full MS and MS/MS spectra of compounds **1**–**30**, Figure S2: Tentative fragmentation pathways of some compounds present in the methanol 90% fraction of *C. cowellii*, Figure S3: Growth environment (municipality of Camagüey, Cuba) and flowering branch of *C. cowellii*, Figure S4: NMR data of mixture M-6A (compound **I** and unidentified impurity), Figure S5: NMR data of mixture M-6B (compounds **II** and **III**), Figure S6: NMR data of mixture M-6C (compounds **IV** and **V**), Figure S7: Flash chromatogram of the methanol 90% fraction, Tables S1–S5: First 10 SMART 2.1 results for the compounds of mixtures M-6A, M-6B, and M-6C.

## Sample Availability

Samples of the compounds are not available from the authors.

## References

[1] Fisher M.C., Hawkins N.J., Sanglard D., Gurr S.J. (2018). Worldwide emergence of resistance to antifungal drugs challenges human health and food security. Science.

[2] Enoch D.A., Yang H., Aliyu S.H., Micallef C. (2017). The Changing Epidemiology of Invasive Fungal Infections. Hum. Fungal Pathog. Identif..

[3] Brown G.D., Denning D.W., Gow N.A.R., Levitz S.M., Netea M.G., White T.C. (2012). Hidden killers: Human fungal infections. Sci. Transl. Med..

[4] Pfaller M.A., Diekema D.J., Turnidge J.D., Castanheira M., Jones R.N. (2019). Twenty Years of the SENTRY Antifungal Surveillance Program: Results for *Candida* Species from 1997–2016. Open Forum Infect. Dis..

[5] de Almeida R.F.M., Santos F.C., Marycz K., Alicka M., Krasowska A., Suchodolski J., Panek J.J., Jezierska A., Starosta R. (2019). New diphenylphosphane derivatives of ketoconazole are promising antifungal agents. Sci. Rep..

[6] Lee Y., Puumala E., Robbins N., Cowen L.E. (2020). Antifungal Drug Resistance: Molecular Mechanisms in *Candida albicans* and beyond. Chem. Rev..

[7] Newman D.J., Cragg G.M. (2020). Natural Products as Sources of New Drugs over the Nearly Four Decades from 01/1981 to 09/2019. J. Nat. Prod..

[8] Méndez D., Escalona-Arranz J.C., Foubert K., Matheeussen A., Van der Auwera A., Piazza S., Cuypers A., Cos P., Pieters L. (2021). Chemical and Pharmacological Potential of *Coccoloba cowellii*, an Endemic Endangered Plant from Cuba. Molecules.

[9] Vukics V., Guttman A. (2008). Structural characterization of flavonoid glycosides by multi-stage mass spectrometry. Mass Spectrom. Rev..

[10] Morreel K., Dima O., Kim H., Lu F., Niculaes C., Vanholme R., Dauwe R., Goeminne G., Inzé D., Messens E. (2010). Mass Spectrometry-Based Sequencing of Lignin Oligomers. Plant Physiol..

[11] Morreel K., Kim H., Lu F., Dima O., Akiyama T., Vanholme R., Niculaes C., Goeminne G., Inze D., Messens E. (2010). Mass Spectrometry-Based Fragmentation as an Identification Tool in Lignomics. Anal. Chem..

[12] Kiyota E., Mazzafera P., Sawaya A.C.H.F. (2012). Analysis of Soluble Lignin in Sugarcane by Ultrahigh Performance Liquid Chromatography—Tandem Mass Spectrometry with a Do-It- Yourself Oligomer Database. Anal. Chem..

[13] Zhang J.-Y., Wang F., Zhang H., Lu J.-Q., Qiao Y.-J. (2014). Rapid Identification of Polymethoxylated Flavonoids in Traditional Chinese Medicines with a Practical Strategy of Stepwise Mass Defect Filtering Coupled to Diagnostic Product Ions Analysis based on a Hybrid LTQ-Orbitrap Mass Spectrometer. Phytochem. Anal..

[14] El-Kawe B.M.A. (2019). A Pharmacognostical Study of Coccoloba peltata Schott Family Polygonaceae.

[15] Nafady A., Ibraheim Z., Abd El-kader A., Ahmed A. (2013). Xanthone and lignan glycosides from the aerial parts of Polygonum bellardii all growing in Egypt. Pharmacogn. Mag..

[16] Lei J., Yao N., Wang K.W. (2013). Phytochemical and chemotaxomic study on *Polygonum perfoliatum* L. Biochem. Syst. Ecol..

[17] Wang K.W., Zhu J.R., Shen L.Q. (2013). A new lignan with anti-tumour activity from *Polygonum perfoliatum* L. Nat. Prod. Res..

[18] Cong H.J., Zhang S.W., Zhang C., Huang Y.J., Xuan L.J. (2012). A novel dimeric procyanidin glucoside from *Polygonum aviculare*. Chin. Chem. Lett..

[19] Rokaya M.B., Münzbergová Z., Timsina B., Bhattarai K.R. (2012). *Rheum australe* D. Don: A review of its botany, ethnobotany, phytochemistry and pharmacology. J. Ethnopharmacol..

[20] Odonbayar B., Murata T., Batkhuu J., Yasunaga K., Goto R., Sasaki K. (2016). Antioxidant Flavonols and Phenolic Compounds from *Atraphaxis frutescens* and Their Inhibitory Activities against Insect Phenoloxidase and Mushroom Tyrosinase. J. Nat. Prod..

[21] Huang G.H., Gao Y., Wu Z.J., Yang Y., Huang D.D., Chen W.S., Sun L.N. (2015). Chemical constituents from *Polygonum capitatum* Buch-Ham. ex D. Don. Biochem. Syst. Ecol..

[22] Chung H.S., Chang L.C., Lee S.K., Shamon L.A., Van Breemen R.B., Mehta R.G., Farnsworth N.R., Pezzuto J.M., Kinghorn A.D. (1999). Flavonoid constituents of *Chorizanthe diffusa* with potential cancer chemopreventive activity. J. Agric. Food Chem..

[23] Gao Y., Su Y., Yan S., Wu Z., Zhang X., Wang T., Gao X. (2010). Hexaoxygenated Flavonoids from *Pteroxygonum giraldii*. Nat. Prod. Commun..

[24] Xiao H., Rao Ravu R., Tekwani B.L., Li W., Liu W.B., Jacob M.R., Khan S.I., Cai X., Peng C.Y., Khan I.A. (2017). Biological evaluation of phytoconstituents from *Polygonum hydropiper*. Nat. Prod. Res..

[25] Datta B.K., Datta S.K., Rashid M.A., Nash R.J., Sarker S.D. (2000). A sesquiterpene acid and flavonoids from *Polygonum viscosum*. Phytochemistry.

[26] Maury G.L., Rodríguez D.M., Hendrix S., Arranz J.C.E., Boix Y.F., Pacheco A.O., Díaz J.G., Morris-Quevedo H.J., Dubois A.F., Aleman E.I. (2020). Antioxidants in Plants: A Valorization Potential Emphasizing the Need for the Conservation of Plant Biodiversity in Cuba. Antioxidants.

[27] Lallemand J.Y., Duteil M. (1977). 13C n.m.r. spectra of quercetin and rutin. Org. Magn. Reson..

[28] Schwingel L.C., Schwingel G.O., Storch N., Barreto F., Bassani V.L. (2014). 3-O-Methylquercetin from organic *Nicotiana tabacum* L. trichomes: Influence of the variety, cultivation and extraction parameters. Ind. Crops Prod..

[29] Rabesa Z.A., Voirin B. (1979). ouveaux aglycones flavoniques O-methyles derives de la mearnsetine chez *Alluaudia ascendens*. Phytochemistry.

[30] Ayanoglu E., Ulubelen A., Clark W.D., Brown G.K., Kerr R.R., Mabry T.J. (1981). Myricetin and quercetin methyl ethers from *Haplopappus integerrimus* var. Punctatus. Phytochemistry.

[31] Mai L.H., Chabot G.G., Grellier P., Quentin L., Dumontet V., Poulain C., Espindola L.S., Michel S., Vo H.T.B., Deguin B. (2015). Antivascular and anti-parasite activities of natural and hemisynthetic flavonoids from New Caledonian *Gardenia* species (Rubiaceae). Eur. J. Med. Chem..

[32] Fang N., Leidig M., Mabry T.J. (1986). Fifty-one flavonoids from *Gutierrezia microcephala*. Phytochemistry.

[33] Rabesa Z.A., Voirin B. (1980). Deux nouveaux aglycones flavoniques isolés de *Decaryiamada gascariensis*. Phytochemistry.

[34] Alexander B.D., Johnson M.D., Pfeiffer C.D., Jiménez-Ortigosa C., Catania J., Booker R., Castanheira M., Messer S.A., Perlin D.S., Pfaller M.A. (2013). Increasing echinocandin resistance in *Candida glabrata*: Clinical failure correlates with presence of FKS mutations and elevated minimum inhibitory concentrations. Clin. Infect. Dis..

[35] Al Aboody M.S., Mickymaray S. (2020). Anti-Fungal Efficacy and Mechanisms of Flavonoids. Antibiotics.

[36] Wu T., Cheng D., He M., Pan S., Yao X., Xu X. (2014). Antifungal action and inhibitory mechanism of polymethoxylated flavones from *Citrus reticulata* Blanco peel against *Aspergillus niger*. Food Control.

[37] Gadetskaya A.V., Tarawneh A.H., Zhusupova G.E., Gemejiyeva N.G., Cantrell C.L., Cutler S.J., Ross S.A. (2015). Sulfated phenolic compounds from *Limonium caspium*: Isolation, structural elucidation, and biological evaluation. Fitoterapia.

[38] Mangoyi R., Midiwo J., Mukanganyama S. (2015). Isolation and characterization of an antifungal compound 5-hydroxy-7,4′-dimethoxyflavone from *Combretum zeyheri*. BMC Complement. Altern. Med..

[39] Yenjai C., Prasanphen K., Daodee S., Wongpanich V., Kittakoop P. (2004). Bioactive flavonoids from Kaempferia parviflora. Fitoterapia.

[40] Fonzi W.A., Irwin M.Y. (1993). Isogenic strain construction and gene mapping in *Candida albicans*. Genetics.

[41] Ramage G., Robertson S.N., Williams C. (2014). Strength in numbers: Antifungal strategies against fungal biofilms. Int. J. Antimicrob. Agents.

[42] Shahzad M., Sherry L., Rajendran R., Edwards C.A., Combet E., Ramage G. (2014). Utilising polyphenols for the clinical management of *Candida albicans* biofilms. Int. J. Antimicrob. Agents.

[43] Caesar L.K., Cech N.B. (2019). Synergy and antagonism in natural product extracts: When 1 + 1 does not equal 2. Nat. Prod. Rep..

[44] Nothias L.F., Nothias-Esposito M., Da Silva R., Wang M., Protsyuk I., Zhang Z., Sarvepalli A., Leyssen P., Touboul D., Costa J. (2018). Bioactivity-Based Molecular Networking for the Discovery of Drug Leads in Natural Product Bioassay-Guided Fractionation. J. Nat. Prod..

[45] Cushnie T.P.T., Lamb A.J. (2005). Antimicrobial activity of flavonoids. Int. J. Antimicrob. Agents.

[46] de Freitas A.L.D., Kaplum V., Rossi D.C.P., da Silva L.B.R., Melhem M. (2018). de S.C.; Taborda, C.P.; de Mello, J.C.P.; Nakamura, C.V.; Ishida, K. Proanthocyanidin polymeric tannins from *Stryphnodendron adstringens* are effective against *Candida* spp. isolates and for vaginal candidiasis treatment. J. Ethnopharmacol..

[47] Da Silva C.R., De Andrade Neto J.B., De Sousa Campos R., Figueiredo N.S., Sampaio L.S., Magalhães H.I.F., Cavalcanti B.C., Gaspar D.M., De Andrade G.M., Lima I.S.P. (2014). Synergistic effect of the flavonoid catechin, quercetin, or epigallocatechin gallate with fluconazole induces apoptosis in *Candida tropicalis* resistant to fluconazole. Antimicrob. Agents Chemother..

[48] Han Y. (2007). Synergic anticandidal effect of epigallocatechin-O-gallate combined with amphotericin B in a murine model of disseminated candidiasis and its anticandidal mechanism. Biol. Pharm. Bull..

[49] Cos P., Vlietinck A.J., Berghe D.V., Maes L. (2006). Anti-infective potential of natural products: How to develop a stronger in vitro “proof-of-concept”. J. Ethnopharmacol..

[50] de Cremer K., Lanckacker E., Cools T.L., Bax M., de Brucker K., Cos P., Cammue B.P.A., Thevissen K. (2015). Artemisinins, new miconazole potentiators resulting in increased activity against *Candida albicans* biofilms. Antimicrob. Agents Chemother..

