# Supplementary material for: Antifungal Activity of Extracts, Fractions, and Constituents from Coccoloba cowellii Leaves
Source: Pharmaceuticals (Basel). 2021 Sep 10;14(9):917. doi: 10.3390/ph14090917 (PMC8469486; doi:10.3390/ph14090917)
Supplement: Supplementary file 1 [file pharmaceuticals-14-00917-s001.zip › pharmaceuticals-1330525-supplementary.pdf]

pharmaceuticals

Supplementary Materials

Antifungal Activity of Extracts, Fractions, and Constituents from *Coccoloba cowellii* Leaves

Daniel Méndez [1], Julio C. Escalona-Arranz [2,*], Enrique Molina Pérez [1], Kenn Foubert [3], An Matheeussen [4], Emmy Tuenter [3], Ann Cuypers [5], Paul Cos [4] and Luc Pieters [3,*]

[1] Chemistry Department, Faculty of Applied Sciences, University of Camagüey, Carretera de Circunvalación Km 5½, Camagüey 74650, Cuba; daniel.mendez@reduc.edu.cu (D.M.); enrique.molina@reduc.edu.cu (E.M.P)
[2] Pharmacy Department, Faculty of Natural and Exact Sciences, Universidad de Oriente, Avenida Patricio Lumumba s/n, Santiago de Cuba 90500, Cuba
[3] Natural Products & Food Research and Analysis (NatuRA), Department of Pharmaceutical Sciences, University of Antwerp, Universiteitsplein 1, BE-2610 Antwerp, Belgium; kenn.foubert@uantwerpen.be (K.F.); emmy.tuenter@uantwerpen.be (E.T.)
[4] Laboratory of Microbiology, Parasitology and Hygiene (LMPH), Faculty of Pharmaceutical, Biomedical and Veterinary Sciences, University of Antwerp, Universiteitsplein 1, BE-2610 Antwerp, Belgium; an.matheeussen@uantwerpen.be (A.M.); paul.cos@uantwerpen.be (P.C.)
[5] Centre for Environmental Sciences, Campus Diepenbeek, Hasselt University, Agoralaan Building D, BE-3590 Diepenbeek, Belgium; ann.cuypers@uhasselt.be
* Correspondence: jcea@uo.edu.cu (J.C.E-A.); luc.pieters@uantwerpen.be (L.P.)

Citation: Méndez, D.; Escalona-Arranz, J.C.; Pérez, E.M.; Foubert, K.; Matheeussen, A.; Tuenter, E.; Cuypers, A.; Cos, P.; Pieters, L. Antifungal Activity of Extracts, Fractions, and Constituents from *Coccoloba cowellii* Leaves. *Pharmaceuticals* **2021**, *14*, 917. https://doi.org/10.3390/ph14090917

Academic Editors: Dejan Stojković and Marina Sokovic

Received: 23 August 2021
Accepted: 8 September 2021
Published: 10 September 2021

Publisher's Note: MDPI stays neutral with regard to jurisdictional claims in published maps and institutional affiliations.

Copyright: © 2021 by the authors. Licensee MDPI, Basel, Switzerland. This article is an open access article distributed under the terms and conditions of the Creative Commons Attribution (CC BY) license (http://creativecommons.org/licenses/by/4.0/).

Figure S1. Full MS and MS/MS spectra of compounds 1-30.

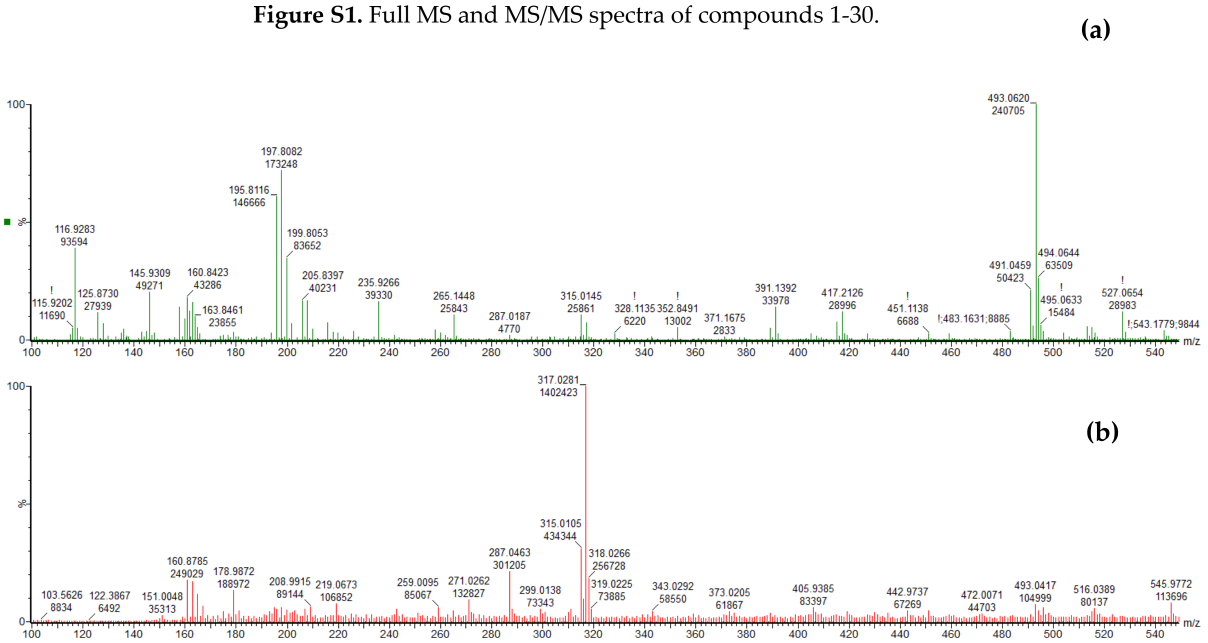

Peak 1 [(a) MS spectrum and (b) MS/MS spectrum], Rt 10.39 min.

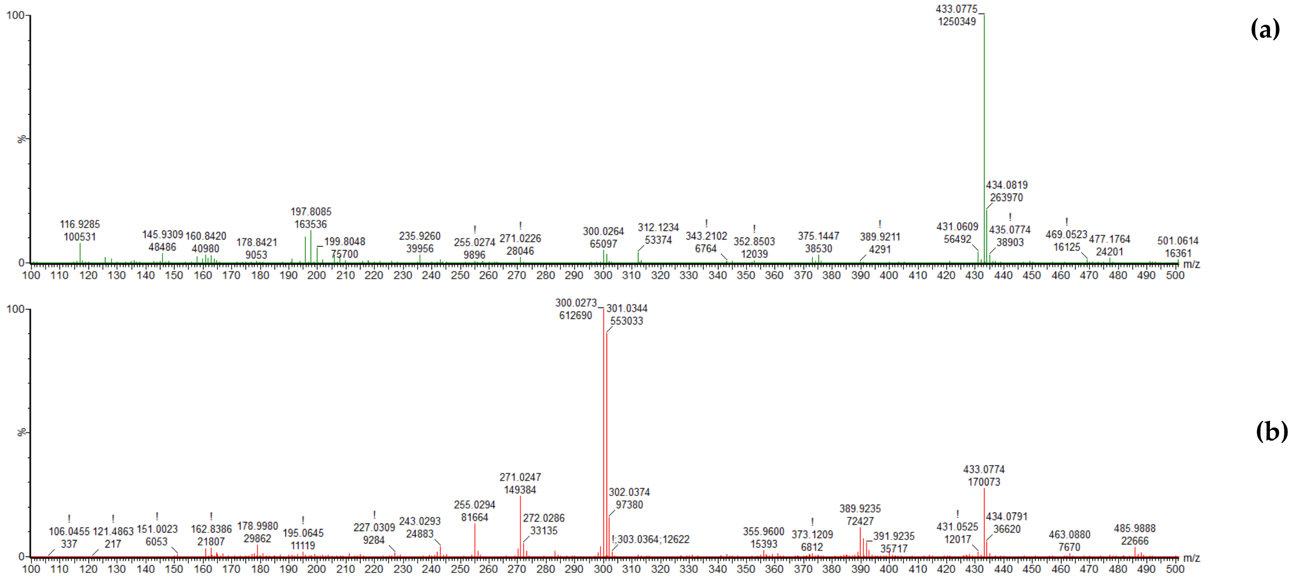

Peak 2 [(a) MS spectrum and (b) MS/MS spectrum], Rt 12.33 min

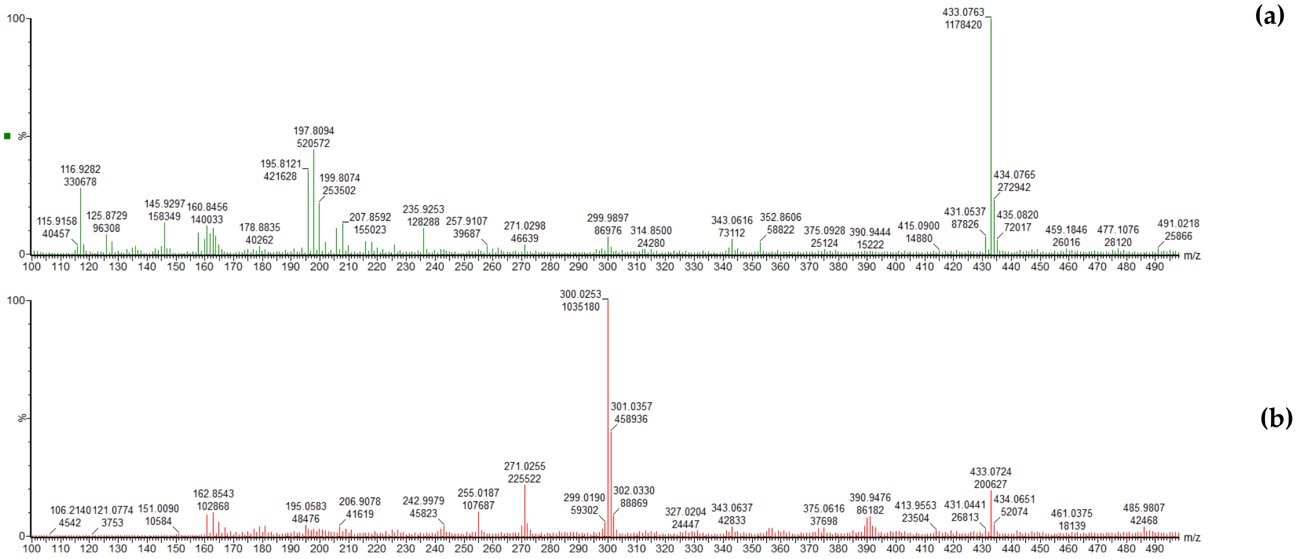

Peak 3 [(a) MS spectrum and (b) MS/MS spectrum], Rt 12.47 min

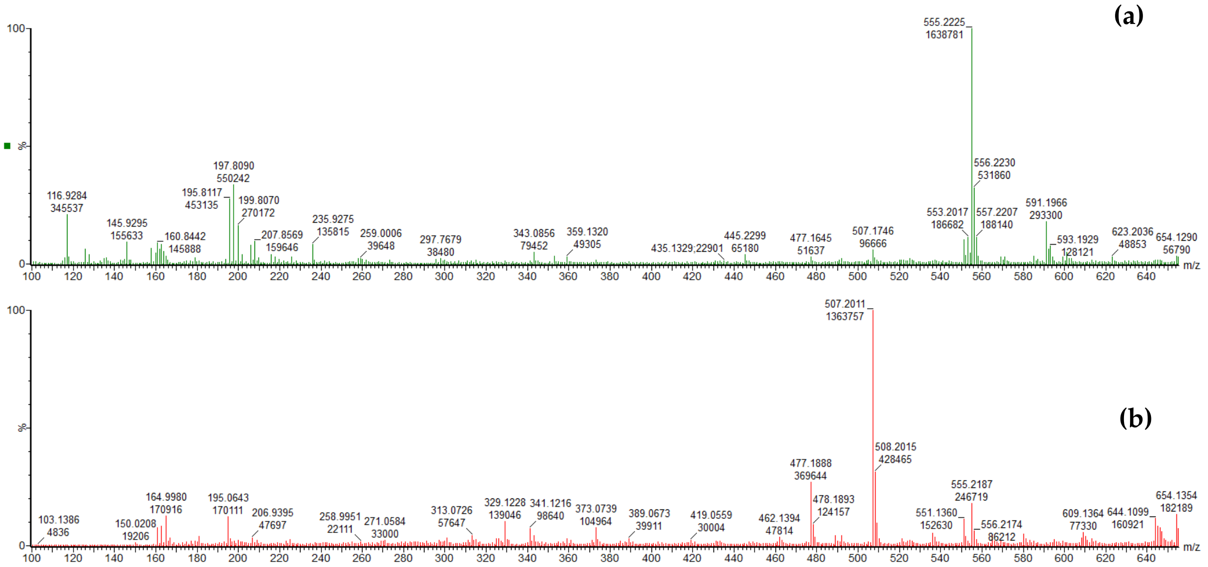

Peak 4 [(a) MS spectrum and (b) MS/MS spectrum], Rt 13.23 min

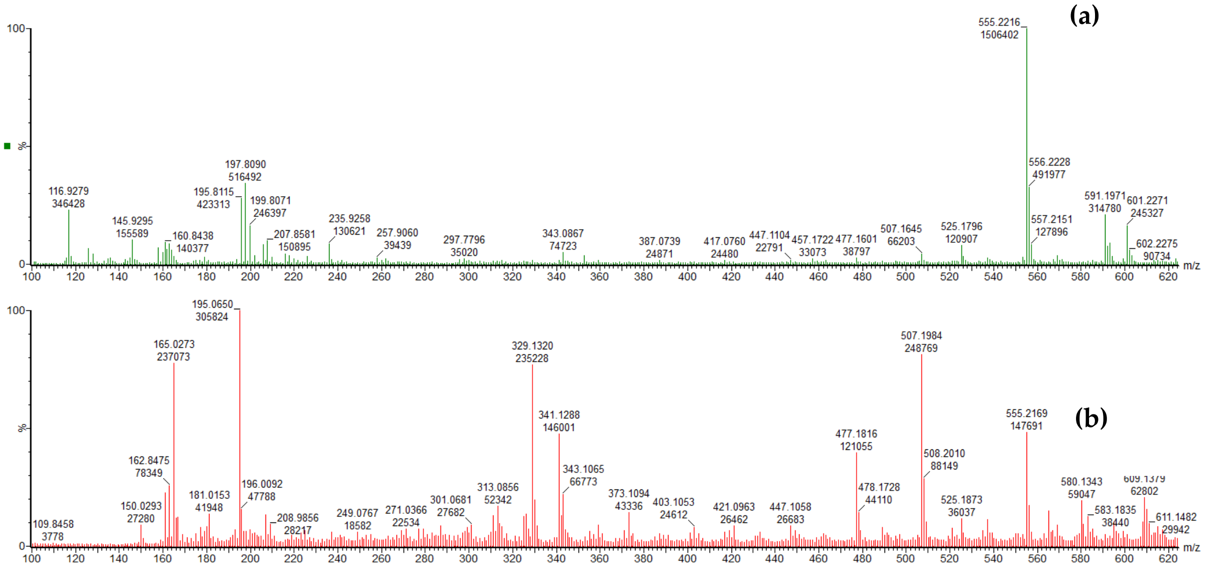

Peak 5 [(a) MS spectrum and (b) MS/MS spectrum], Rt 13.50 min

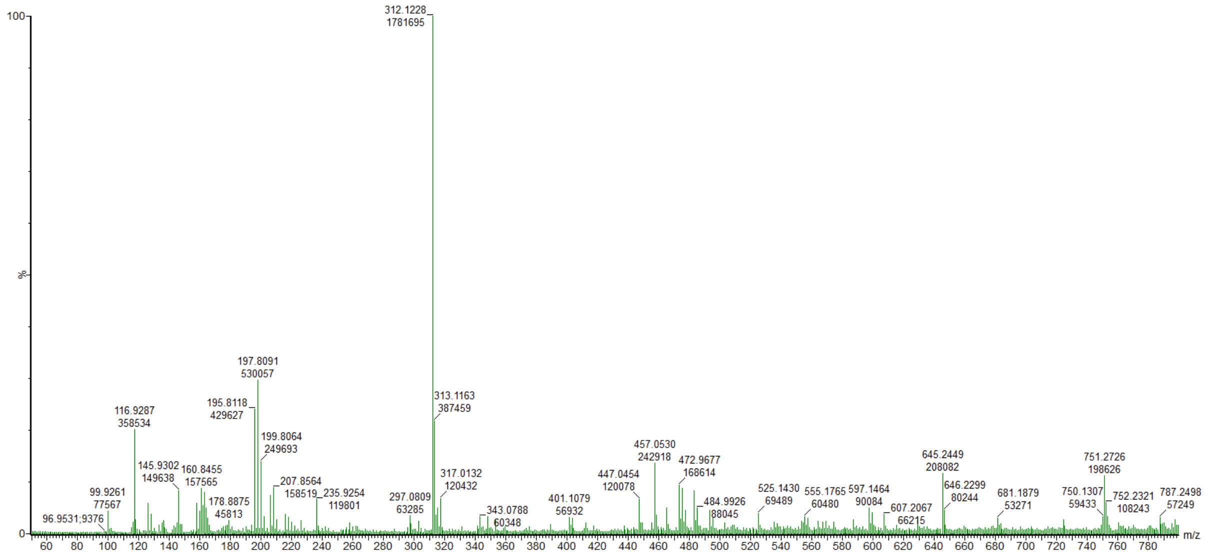

Peak 6 MS spectrum, Rt 13.85 min

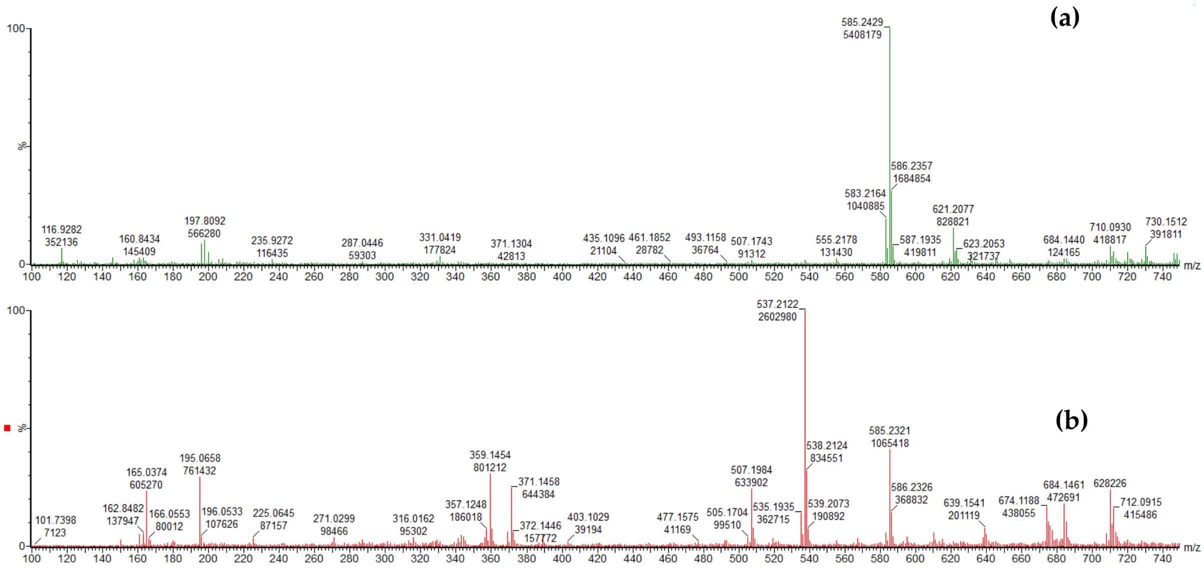

Peak 7 [(a) MS spectrum and (b) MS/MS spectrum], Rt 14.49 min

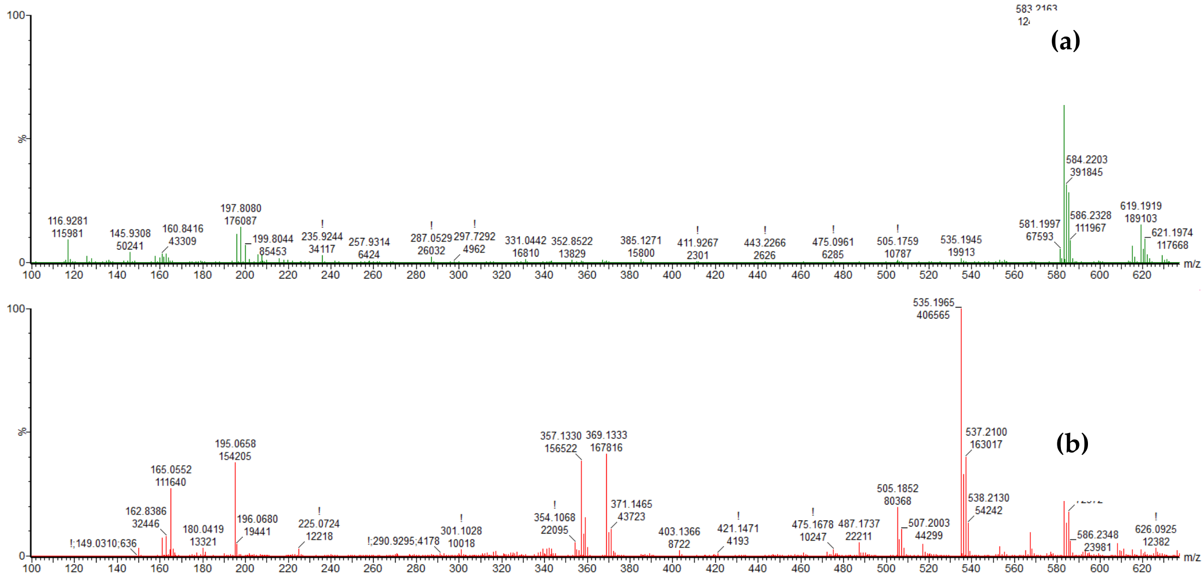

Peak 8 [(a) MS spectrum and (b) MS/MS spectrum], Rt 14.56 min

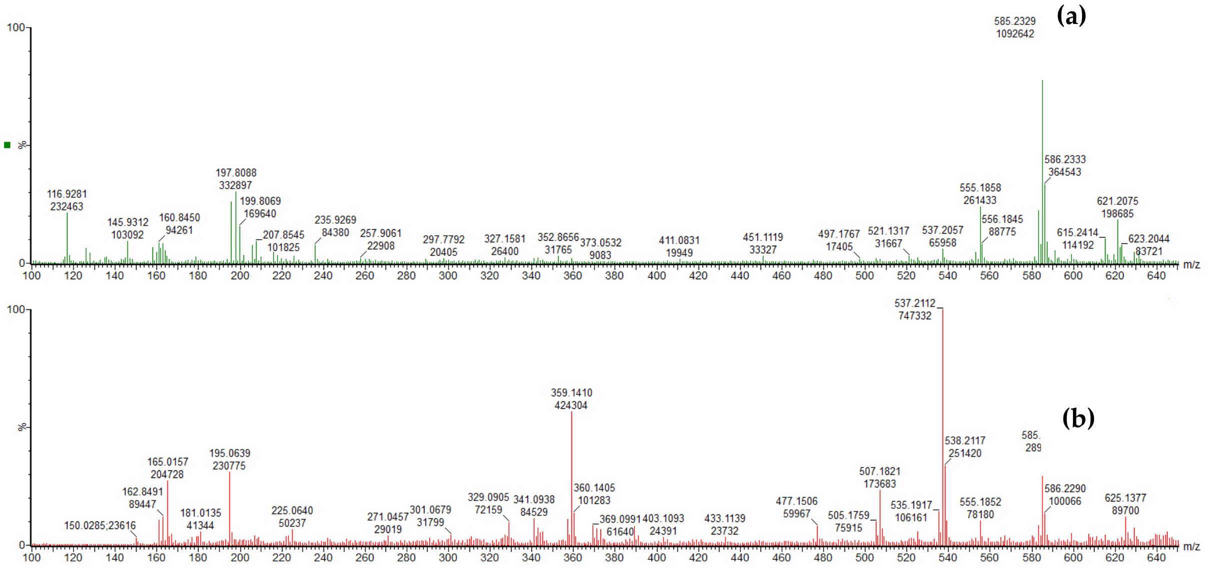

Peak 9 [(a) MS spectrum and (b) MS/MS spectrum], Rt 14.82 min

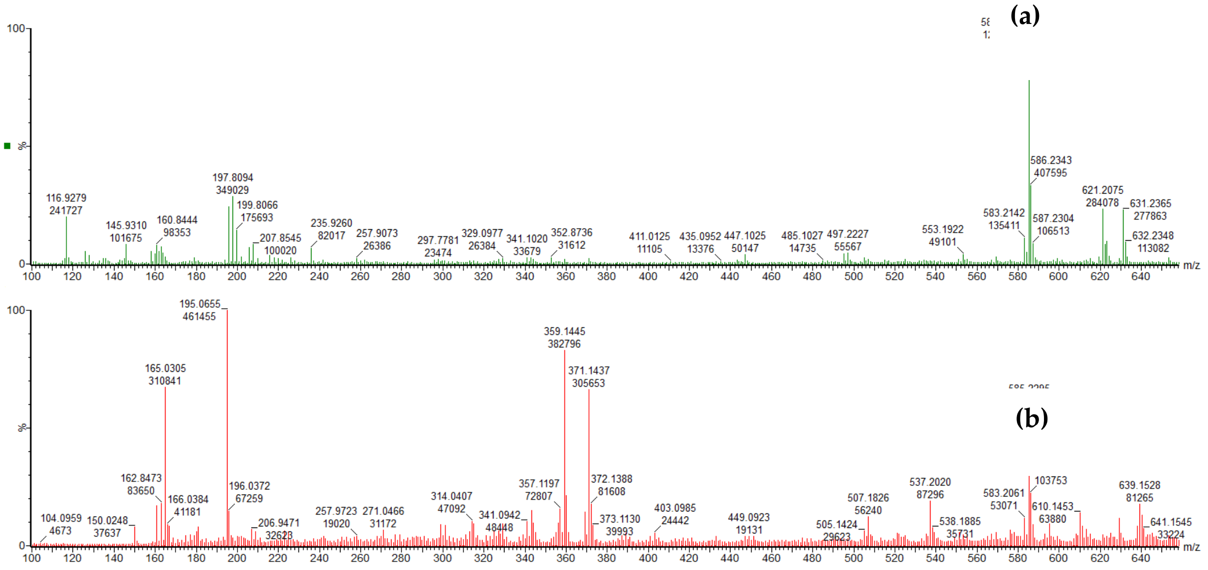

Peak 10 [(a) MS spectrum and (b) MS/MS spectrum], Rt 15.09 min

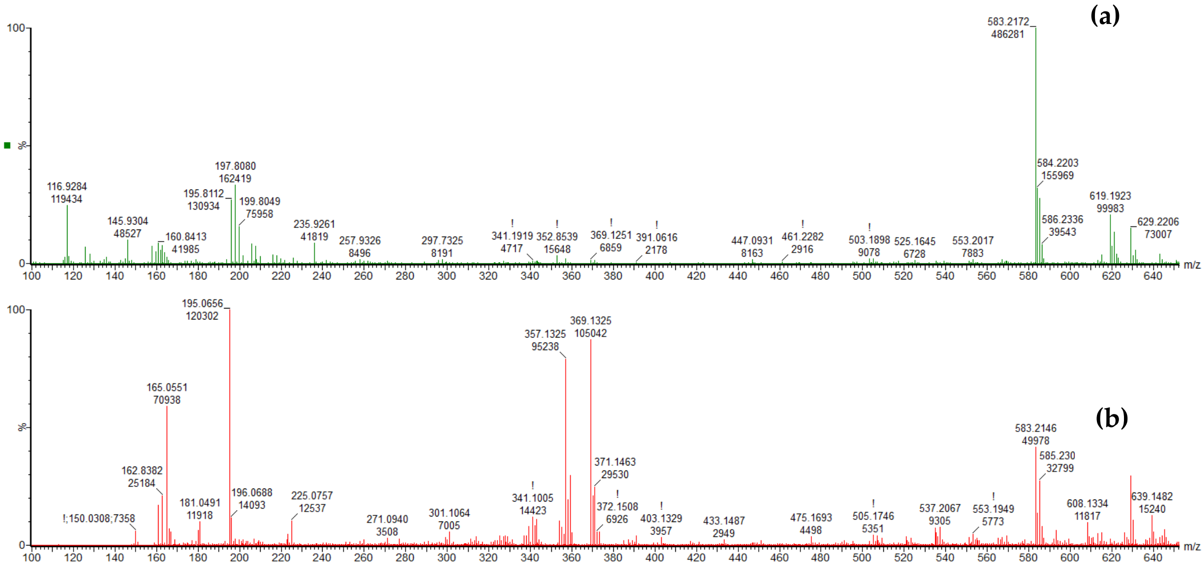

Peak 11 [(a) MS spectrum and (b) MS/MS spectrum], Rt 15.17 min

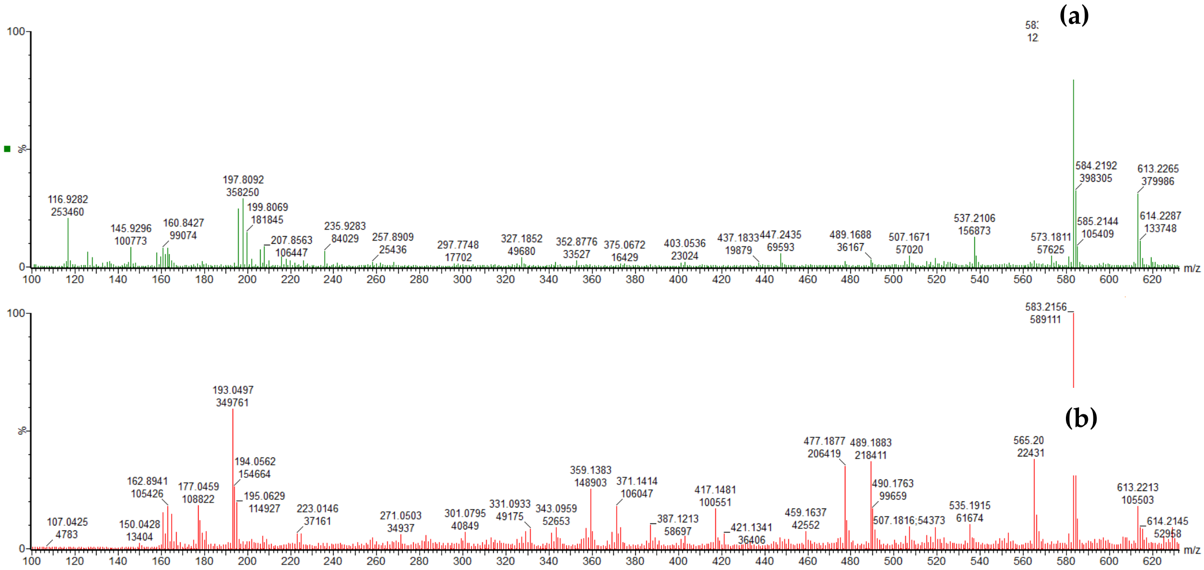

Peak 12 [(a) MS spectrum and (b) MS/MS spectrum], Rt 15.76 min

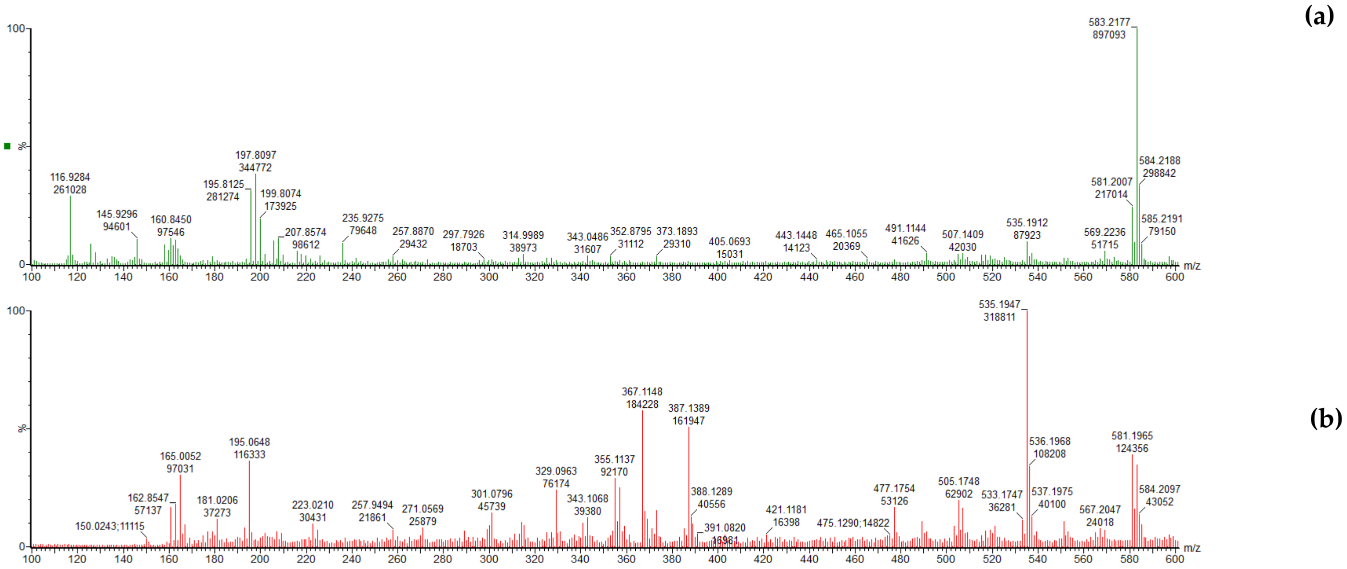

Peak 13 [(a) MS spectrum and (b) MS/MS spectrum], Rt 16.02 min

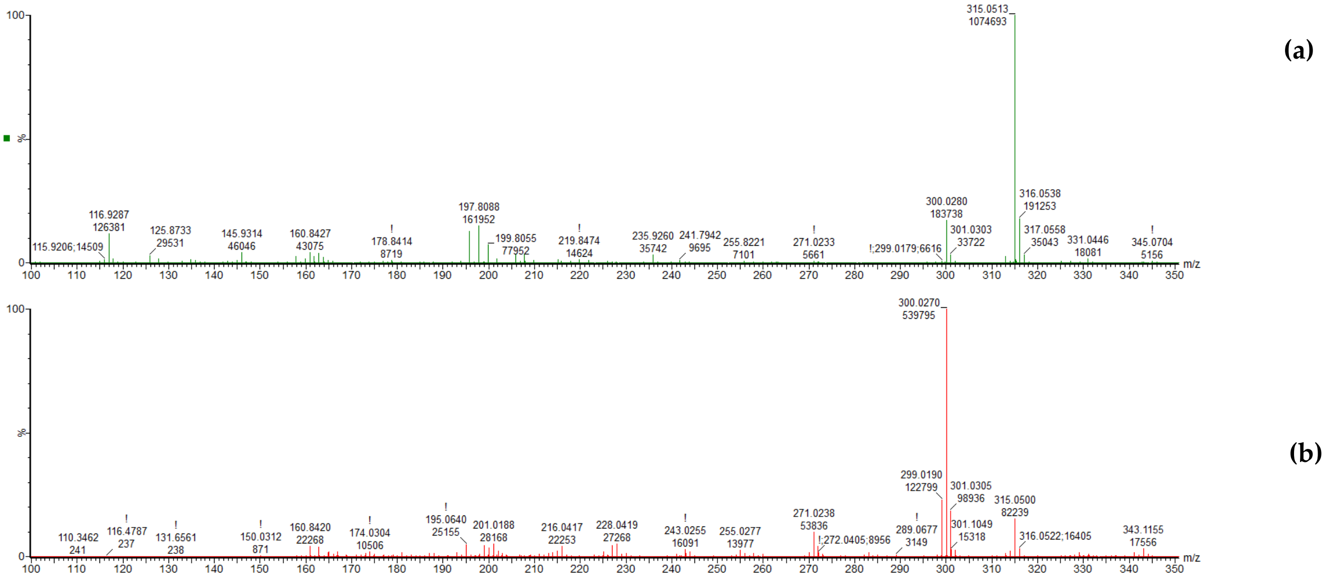

Peak 14 [(a) MS spectrum and (b) MS/MS spectrum], Rt 16.12 min

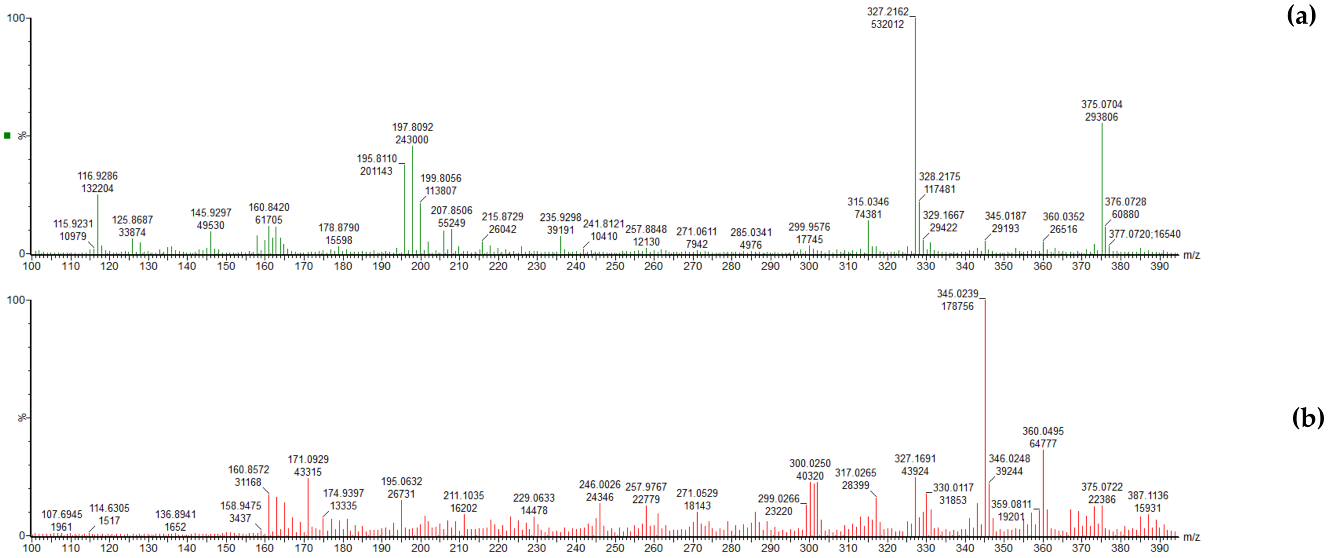

Peak 15 [(a) MS spectrum and (b) MS/MS spectrum], Rt 16.24 min

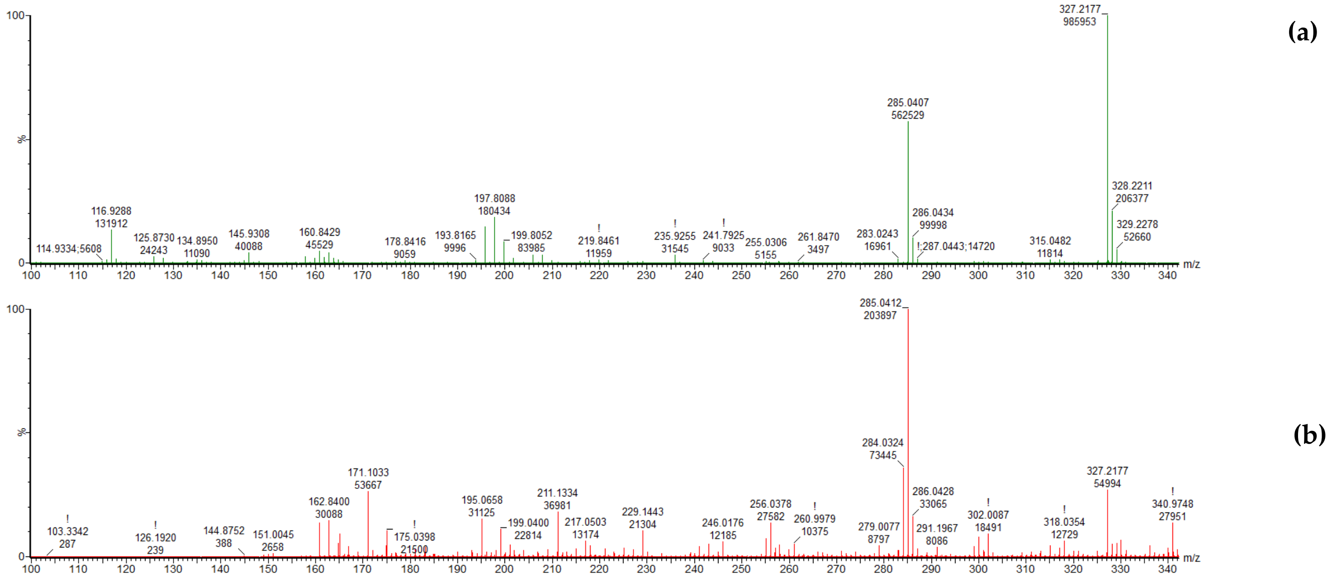

Peak 16 [(a) MS spectrum and (b) MS/MS spectrum], Rt 16.37 min

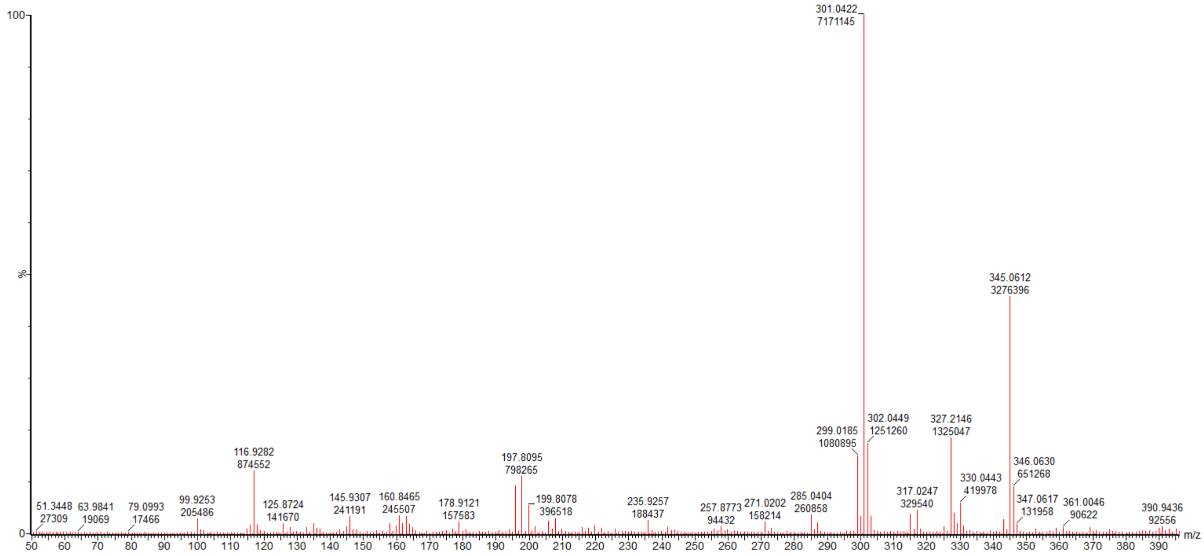

Peak 17 [(a) MS spectrum and (b) MS/MS spectrum], Rt 16.49 min

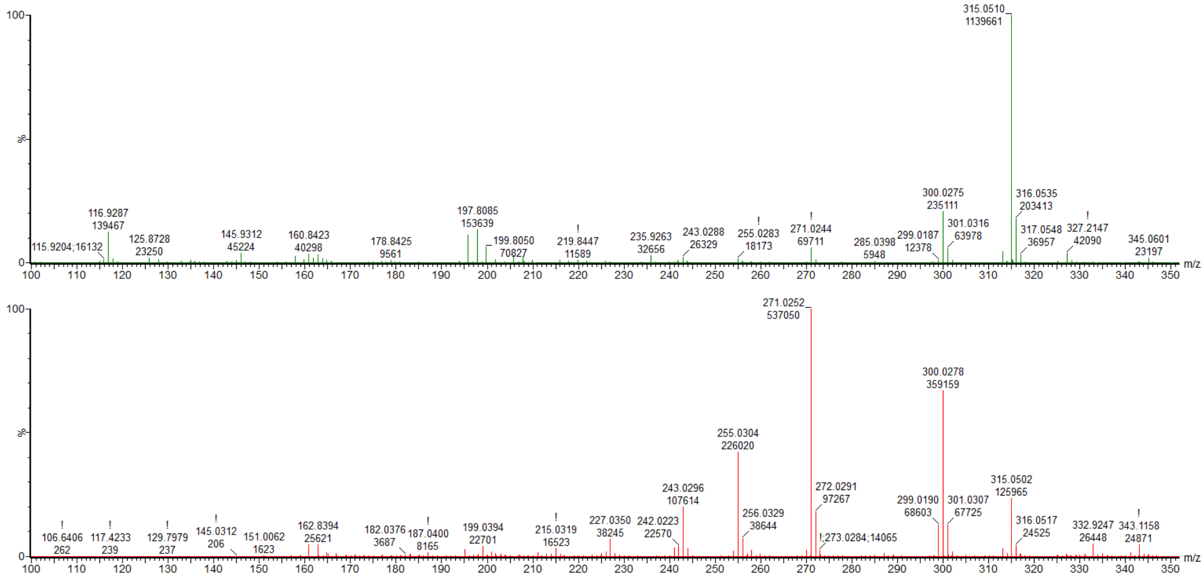

(a)

(b)

Peak 18 [(a) MS spectrum and (b) MS/MS spectrum], Rt 16.71 min

(a)

(b)

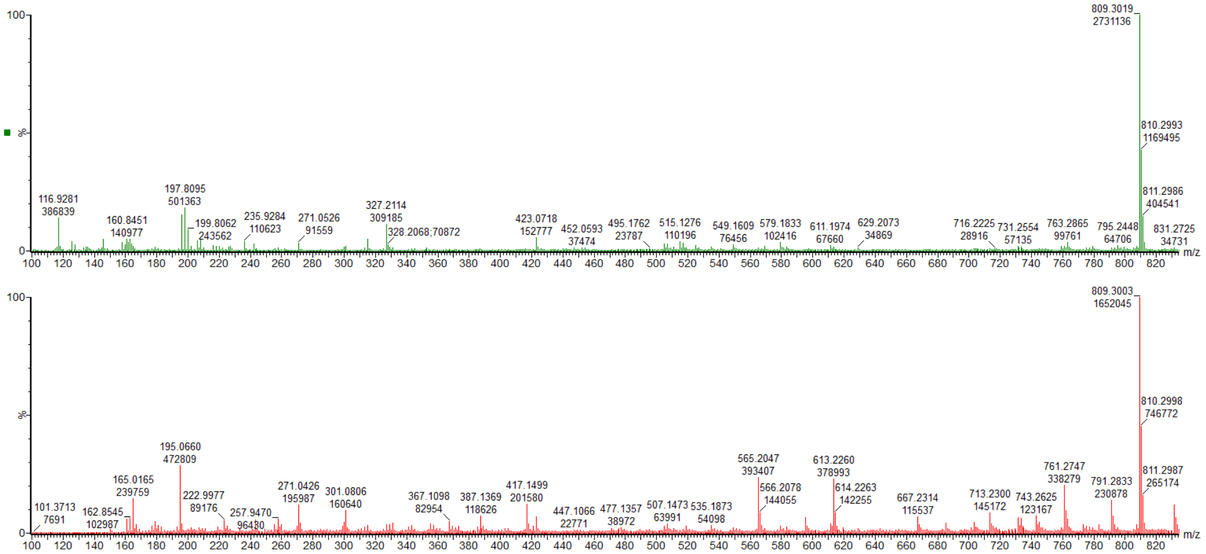

Peak 19 [(a) MS spectrum and (b) MS/MS spectrum], Rt 16.85 min

(a)

(b)

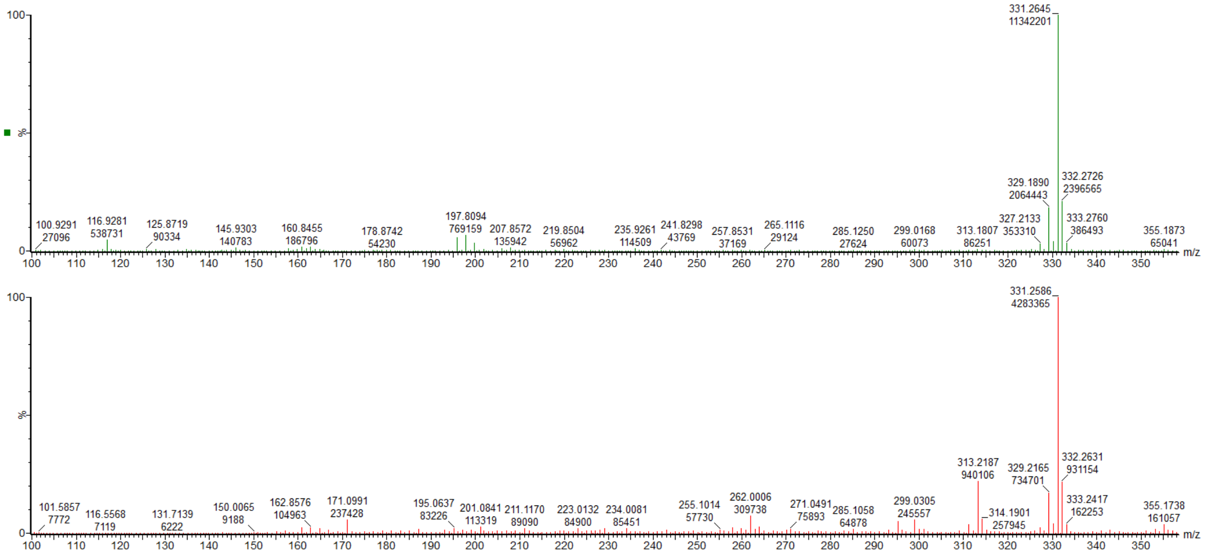

Peak 20 [(a) MS spectrum and (b) MS/MS spectrum], Rt 17.11 min

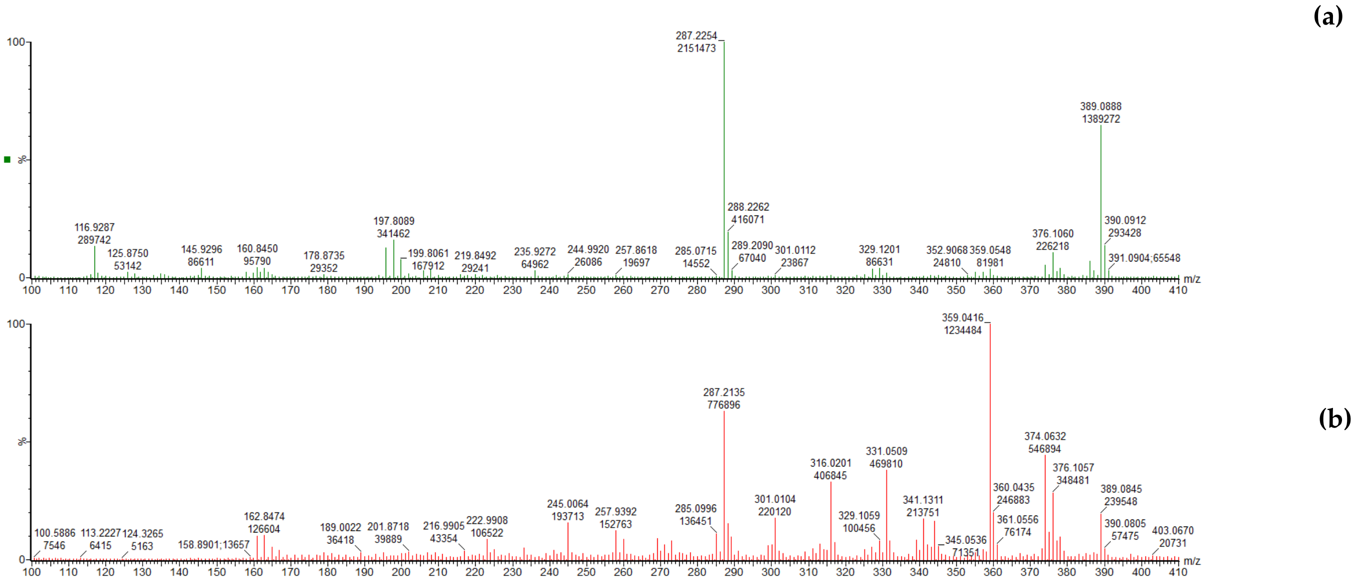

Peak 21 [(a) MS spectrum and (b) MS/MS spectrum], Rt 17.73 min

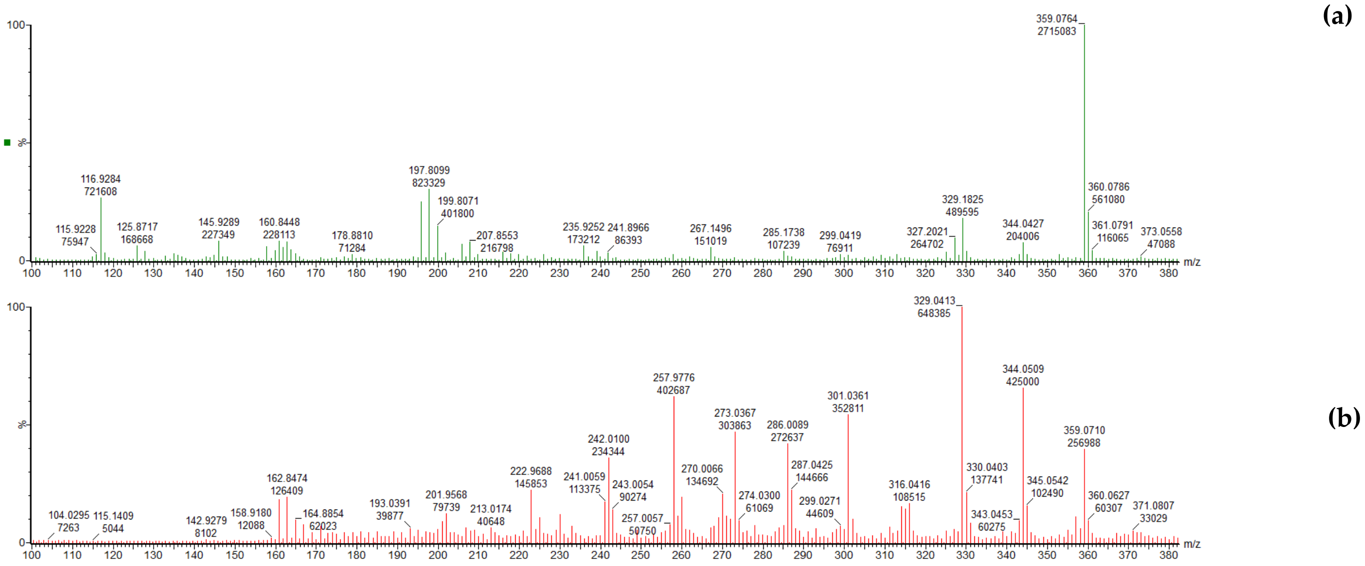

Peak 22 [(a) MS spectrum and (b) MS/MS spectrum], Rt 17.99 min

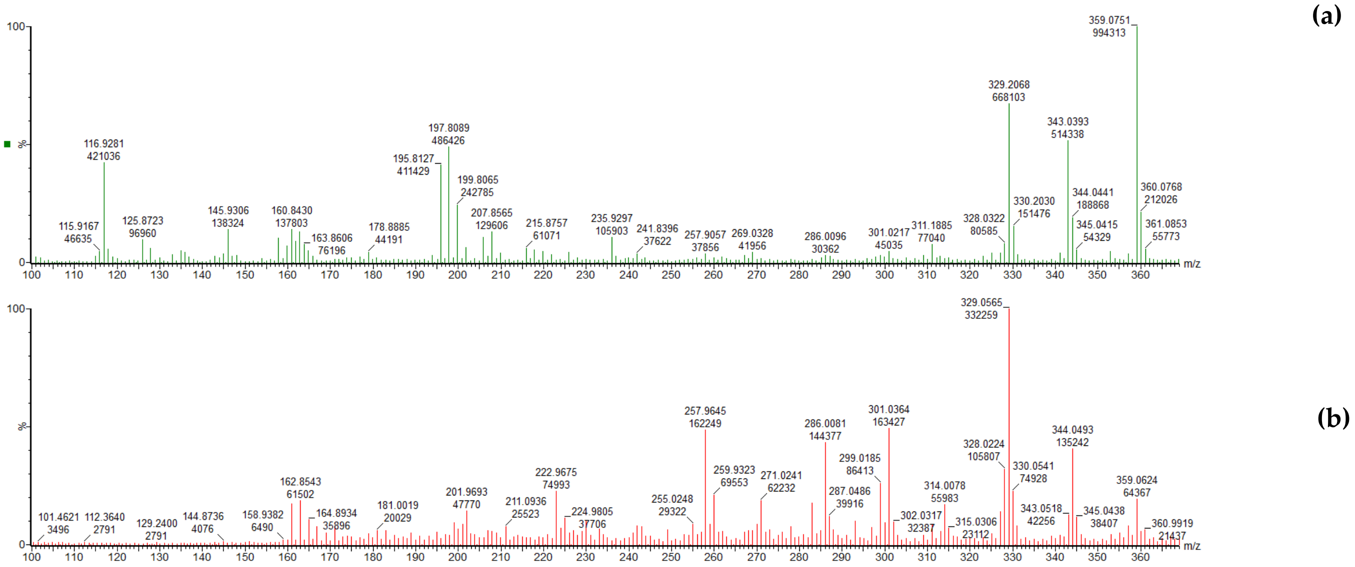

Peak 23 [(a) MS spectrum and (b) MS/MS spectrum], Rt 18.13 min

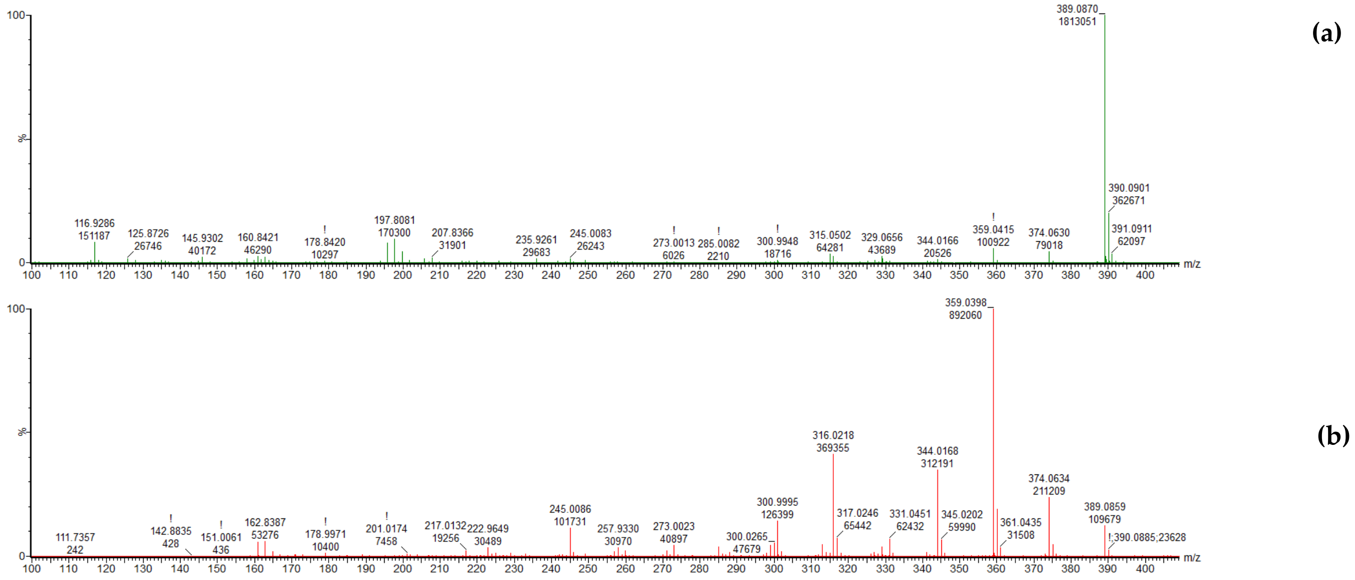

Peak 24 [(a) MS spectrum and (b) MS/MS spectrum], Rt 18.25 min

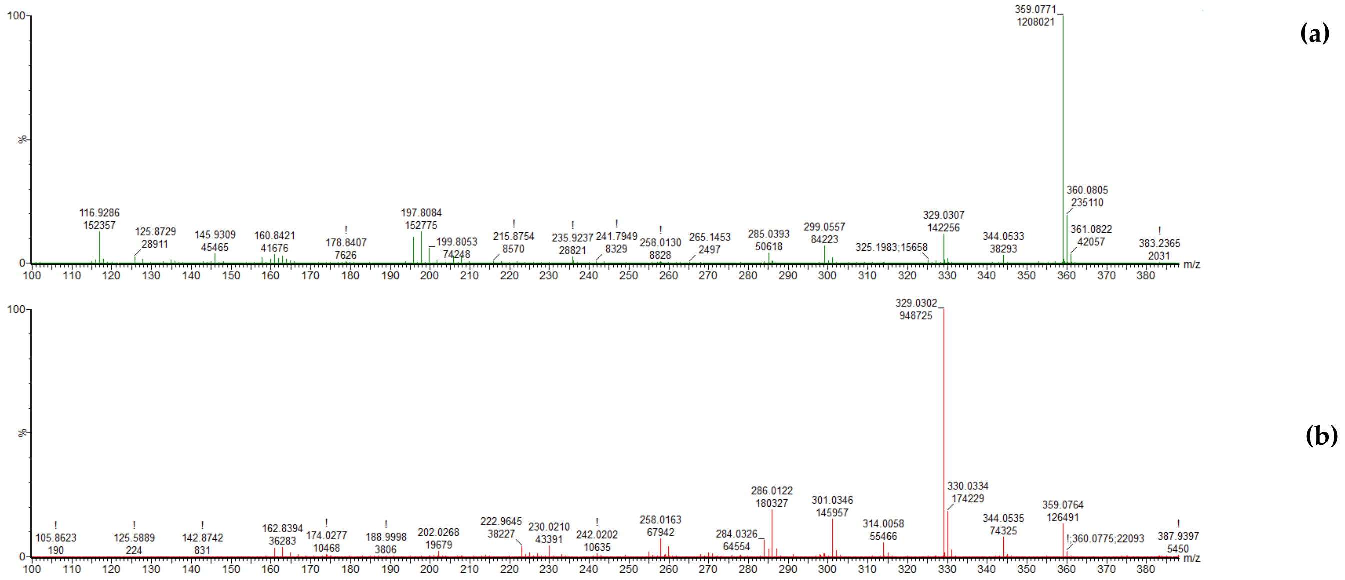

Peak 25 [(a) MS spectrum and (b) MS/MS spectrum], Rt 18.48 min

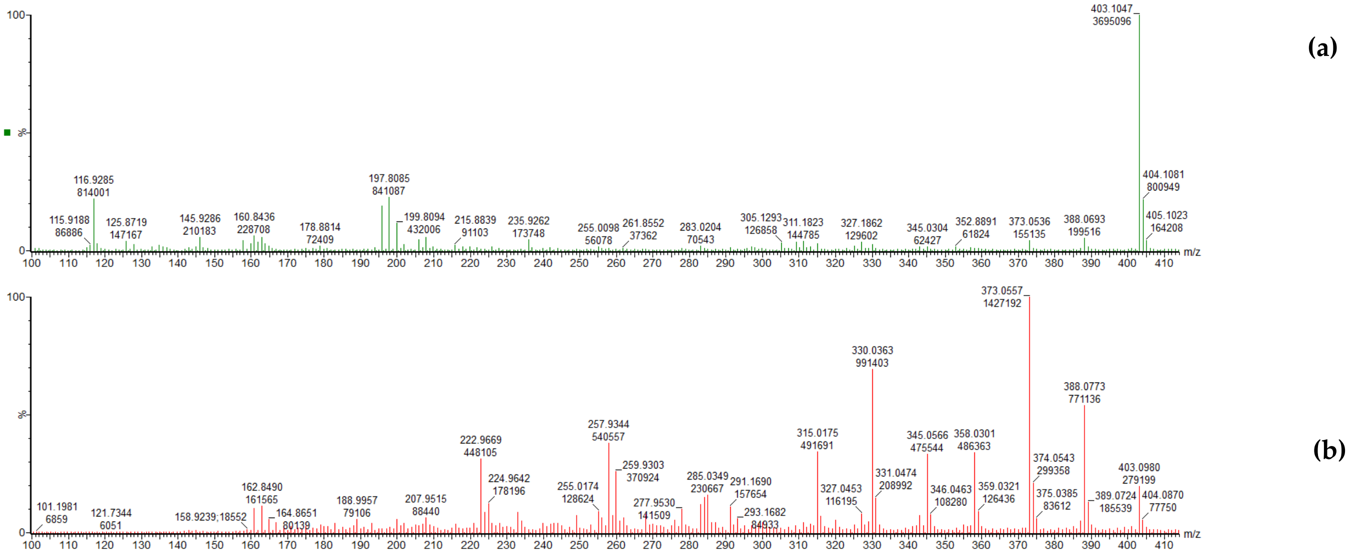

Peak 26 [(a) MS spectrum and (b) MS/MS spectrum], Rt 19.58 min

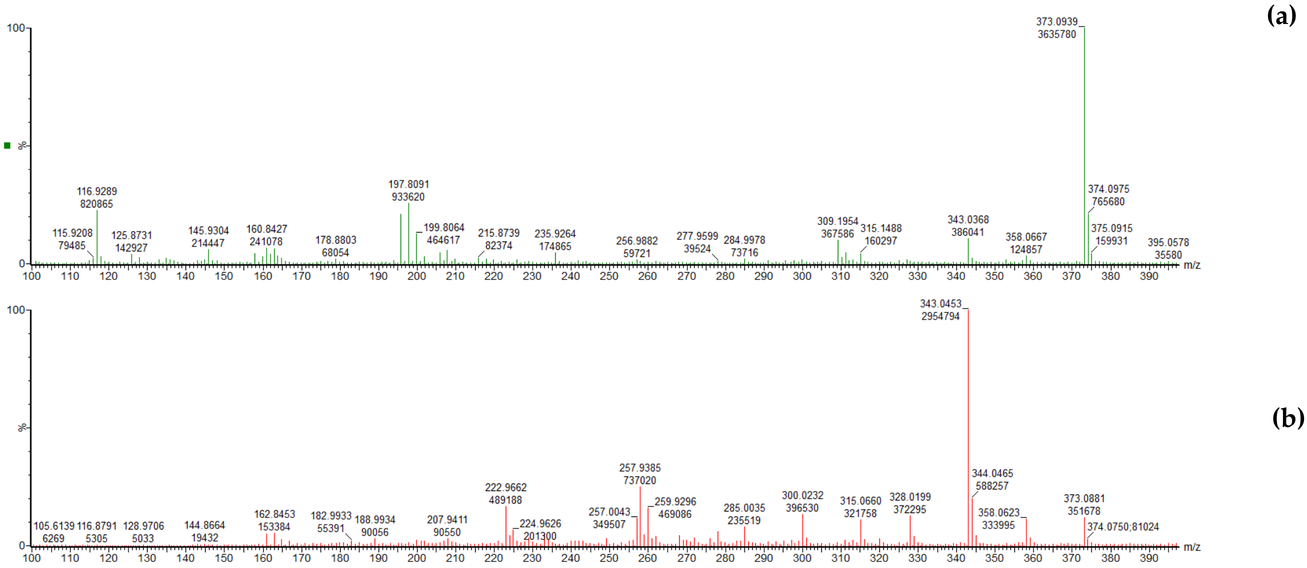

Peak 27 [(a) MS spectrum and (b) MS/MS spectrum], Rt 19.83 min

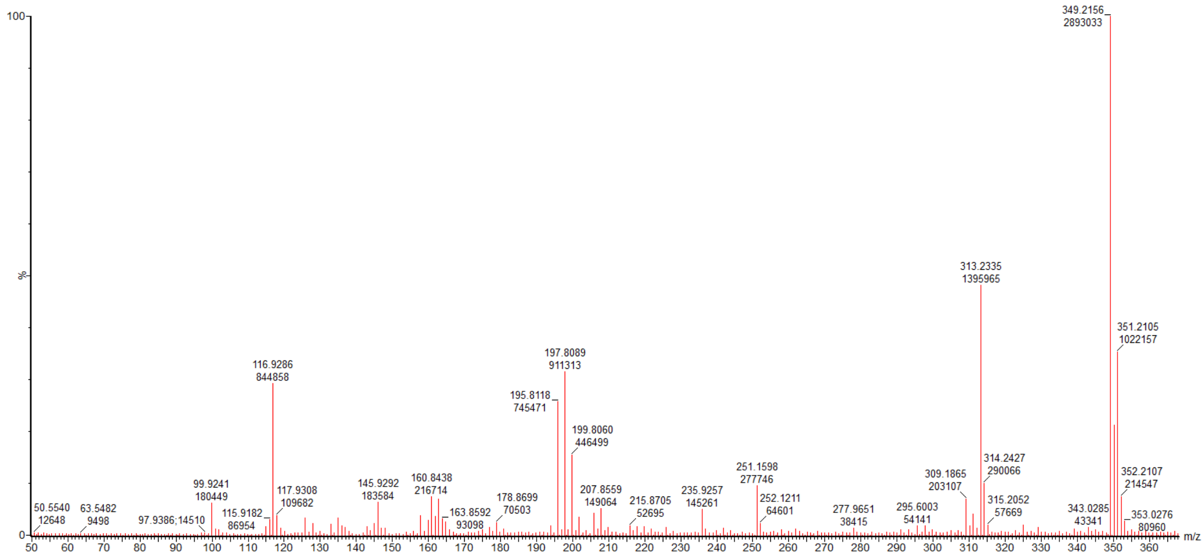

Peak 28 MS spectrum, Rt 20.18 min

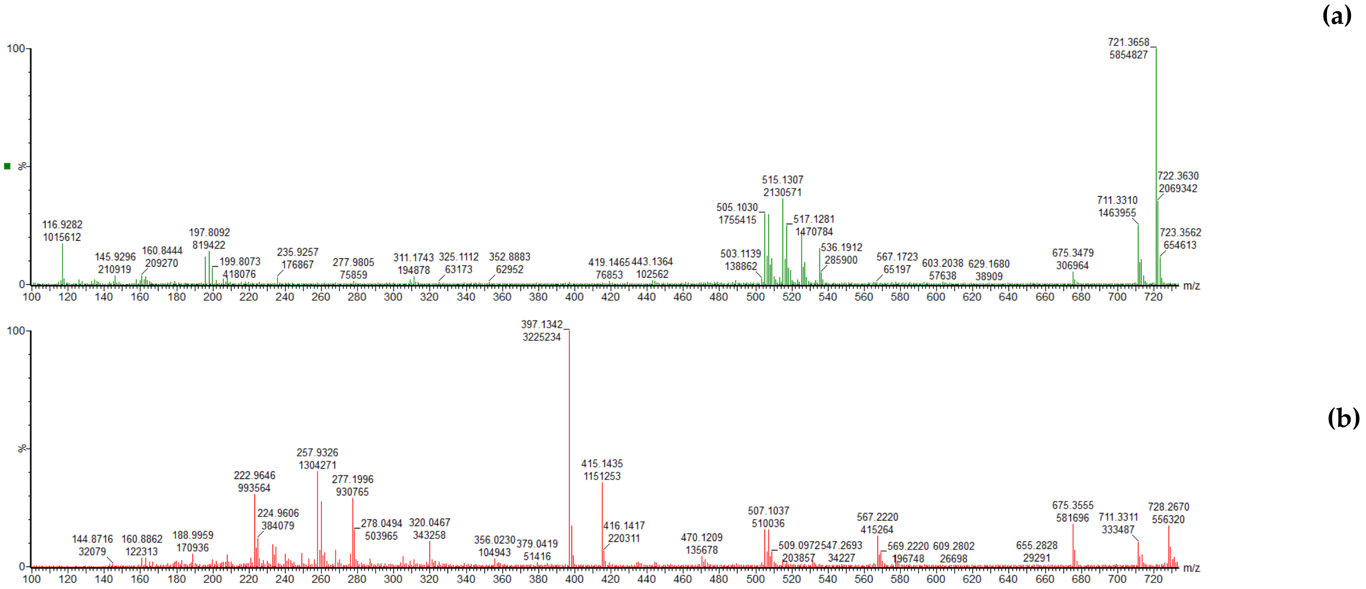

Peak 29 [(a) MS spectrum and (b) MS/MS spectrum], Rt 21.03 min

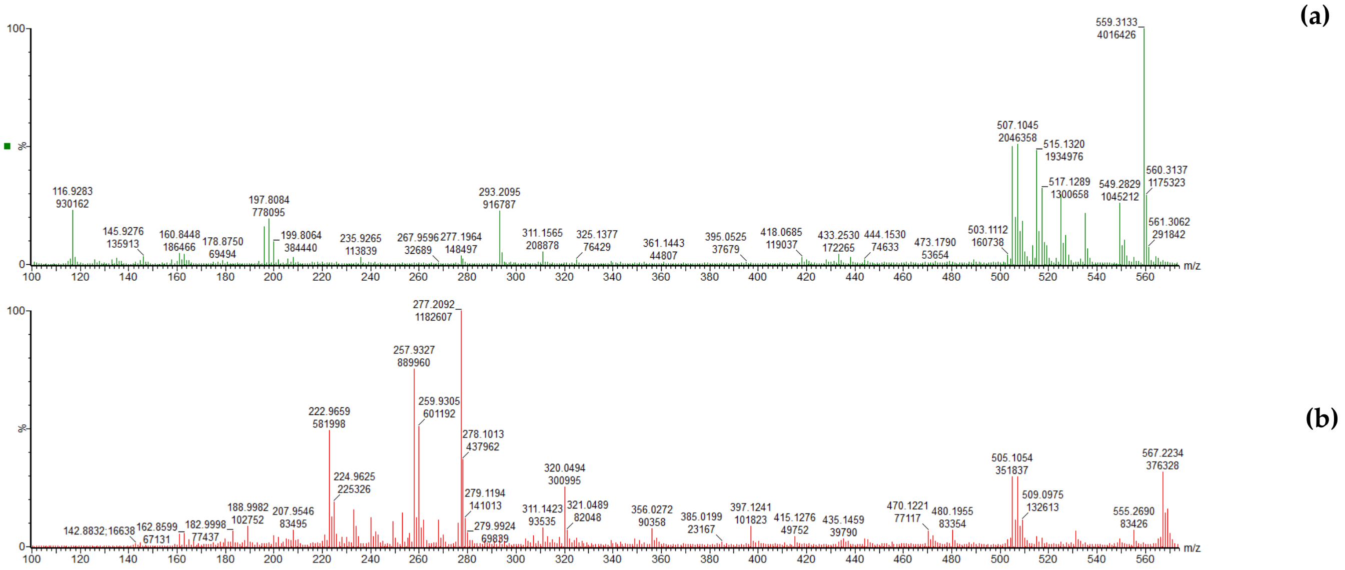

Peak 30 [(a) MS spectrum and (b) MS/MS spectrum], Rt 22.15 min

Figure S2. Tentative fragmentation pathways of some compounds present in the of the methanol 90% fraction of the total extract of *C. cowellii*.

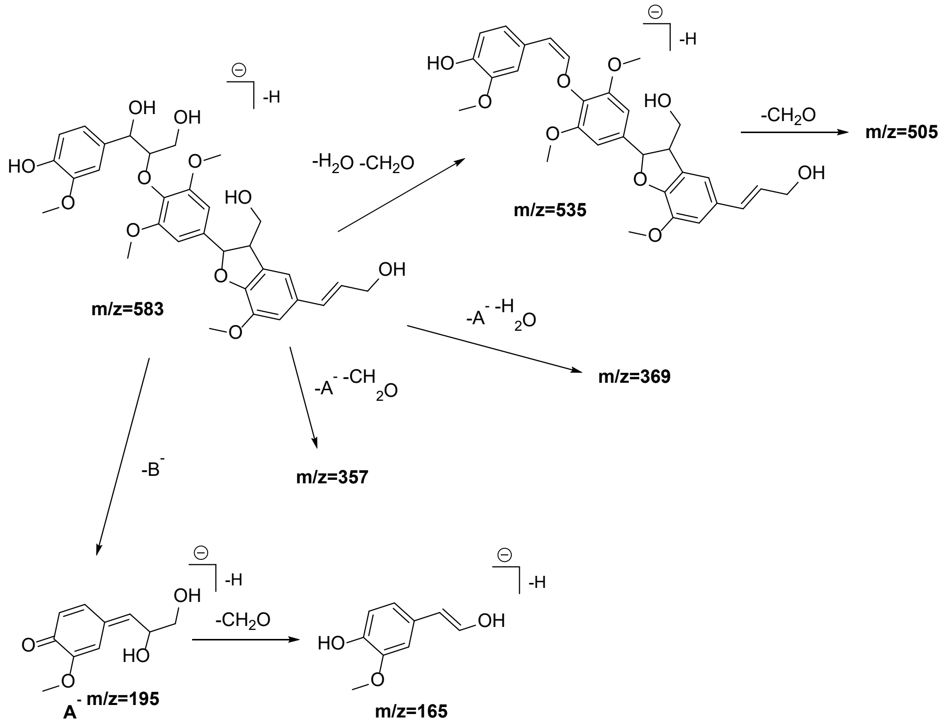

Peak 8, Rt 14.56 min, tentative identification: Trilignol type G(8–O–4)X(8–5)X (being X=either S or G). The represented formula is only for illustrative purpose. Hypothetical fragmentation pattern taken from Morreel et al. [1,2].

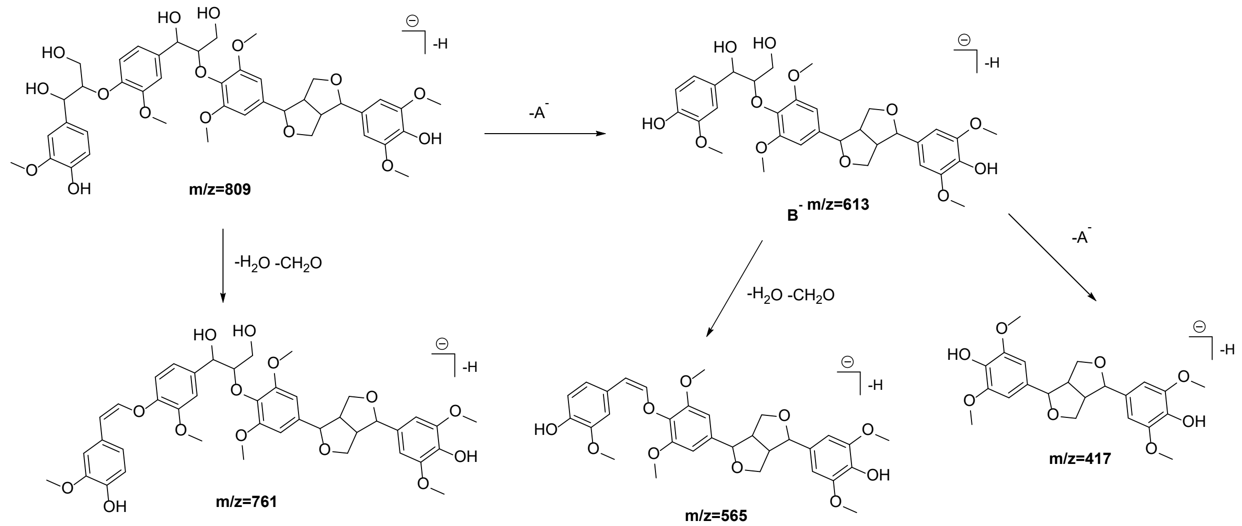

Peak 19, Rt 16.85 min, tentative identification: Tetralignol type G(8–O–4)G(8–O–4)S(8–8)S. The represented formula is only for illustrative purpose. Hypothetical fragmentation pattern taken from Morreel et al. [1,2].

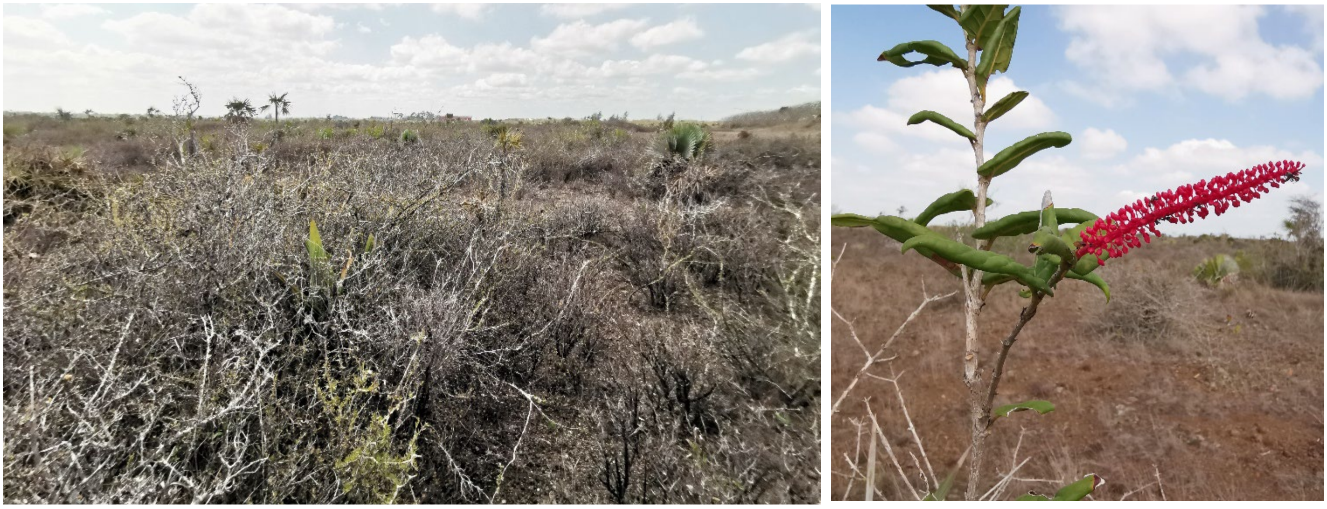

Figure S3. Growth environment (municipality of Camagüey, Cuba) and flowering branch of *C. cowellii*.

Figure S4. NMR data of mixture **M-6A** (compound **I** and unidentified impurity).

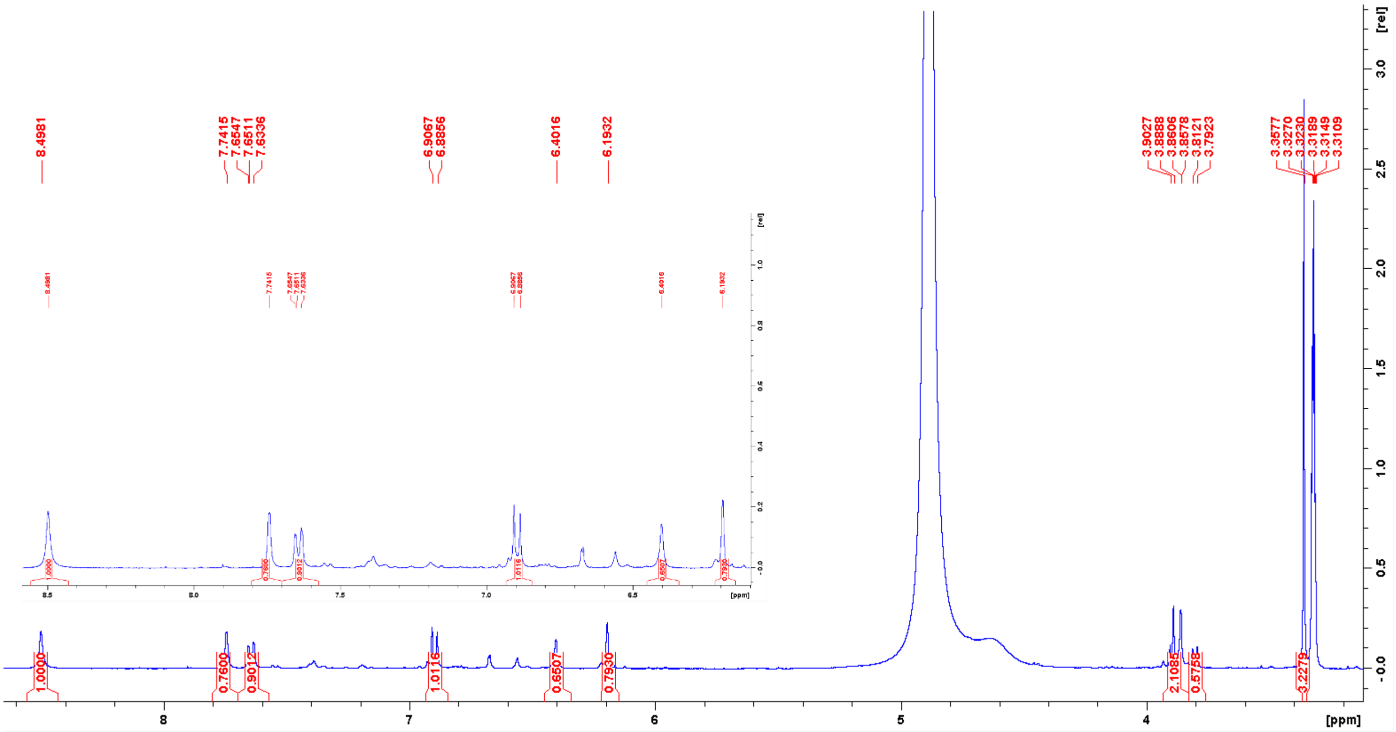

^{1}H NMR spectrum (400 MHz, methanol-d_4)

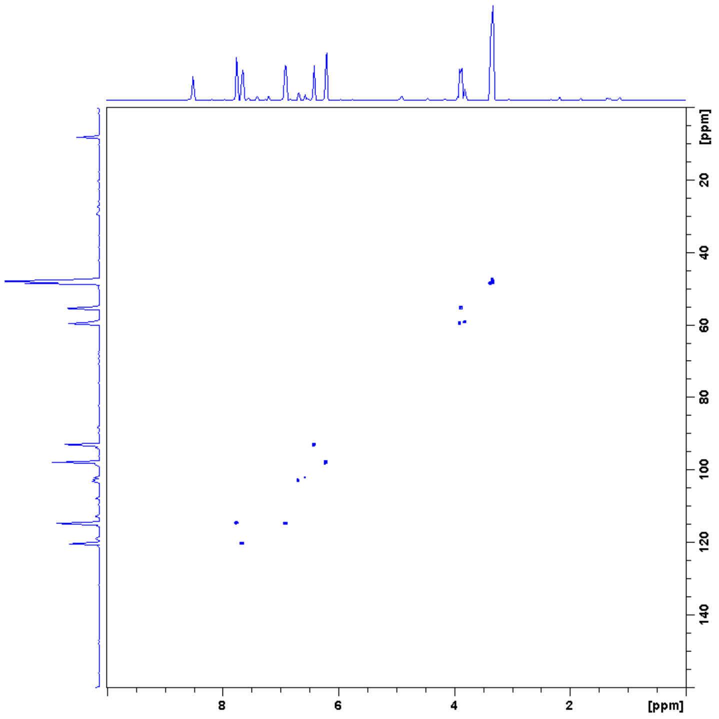

HSQC spectrum (methanol-*d*4)

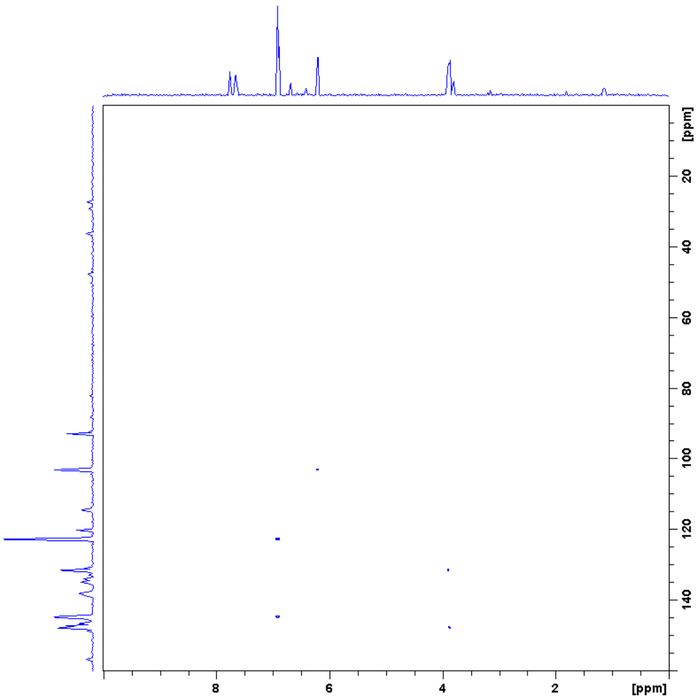

HMBC spectrum (methanol-*d*4)

Figure S5. NMR data of mixture **M-6B** (compounds **II** and **III**).

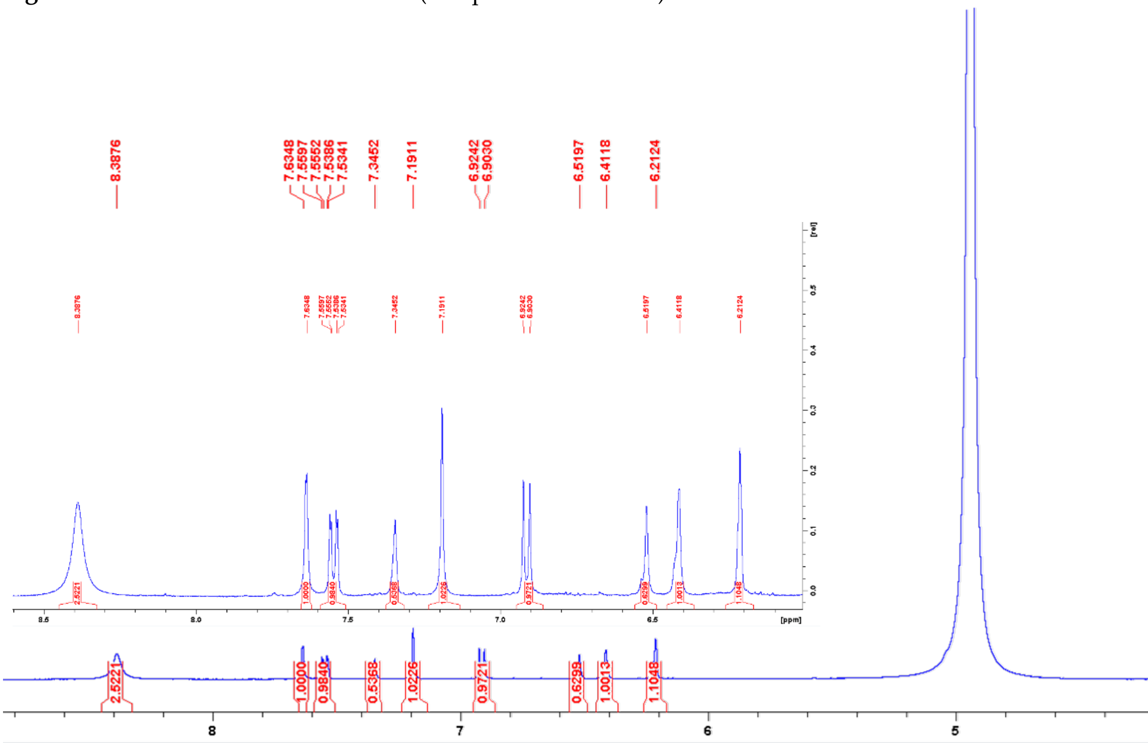

^{1}H NMR spectrum (400 MHz, methanol-d_4)

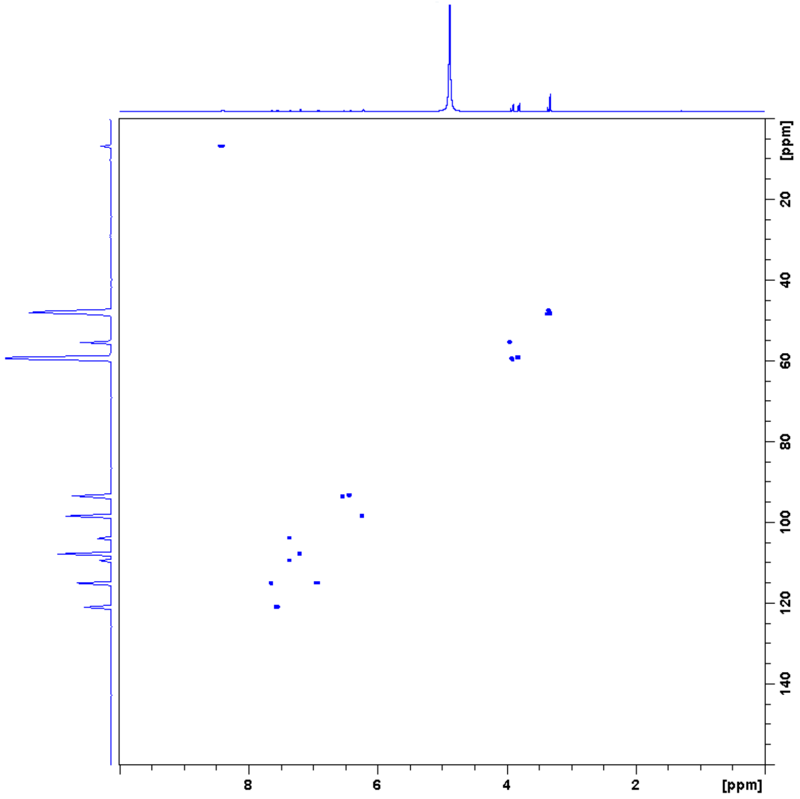

HSQC spectrum (methanol-d_4)

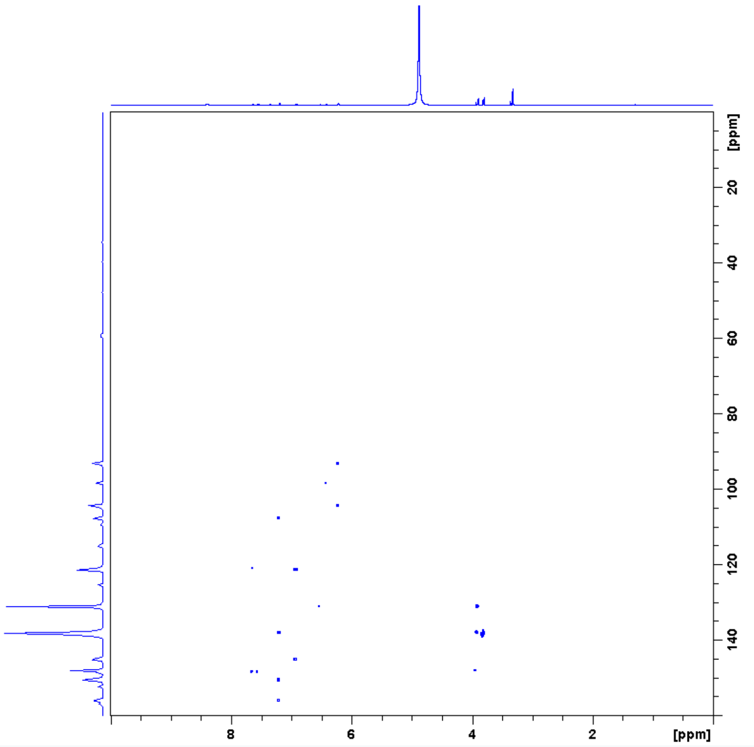

HMBC spectrum (methanol-d_4)

Figure S6. NMR data of mixture **M-6C** (compounds **IV** and **V**).

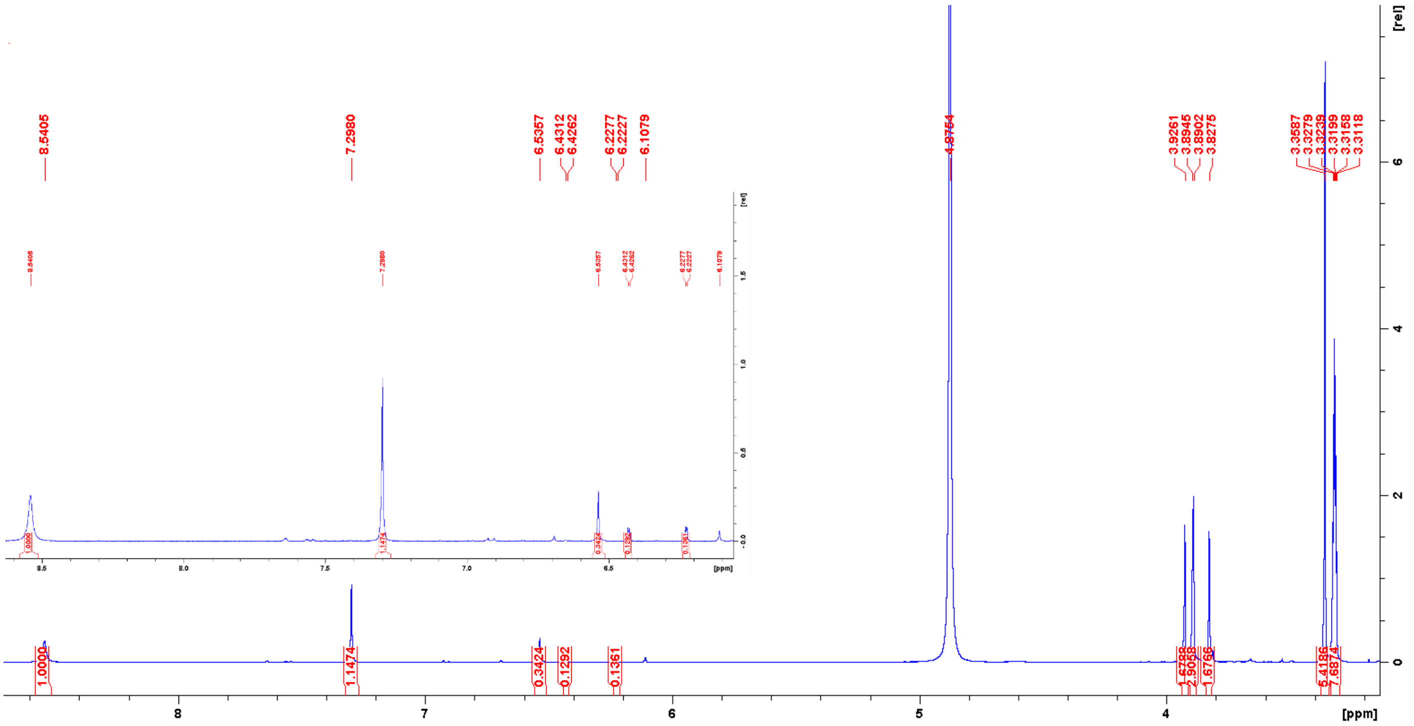

^{1}H NMR spectrum (400 MHz, methanol-d_4)

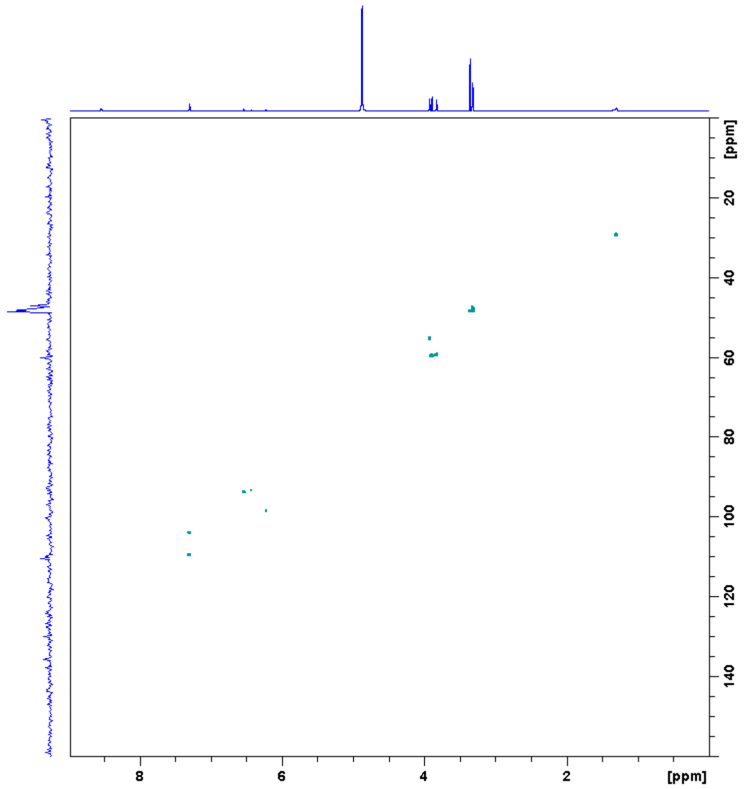

HSQC spectrum (methanol-d_4)

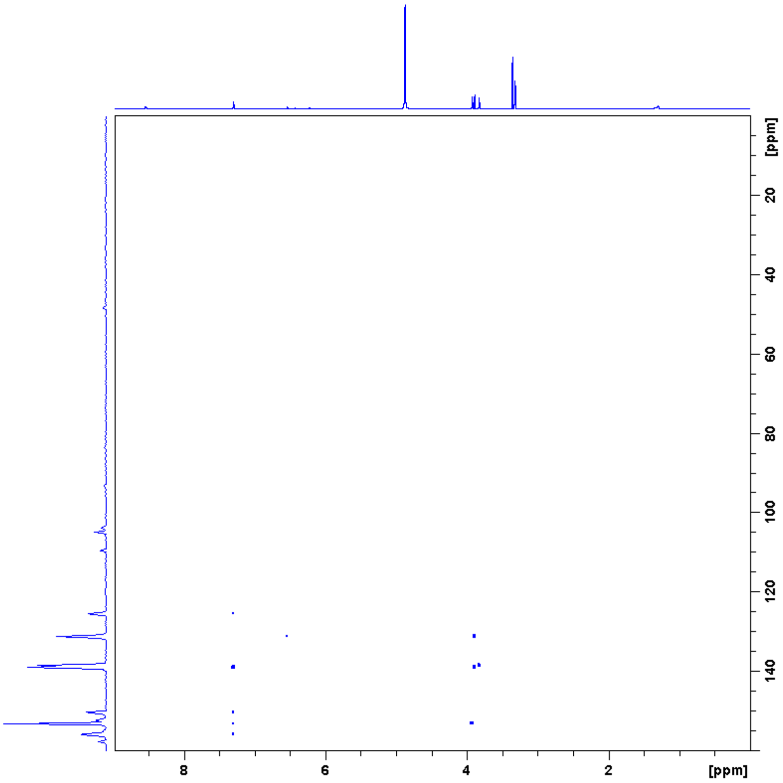

HMBC spectrum (methanol-d_4)

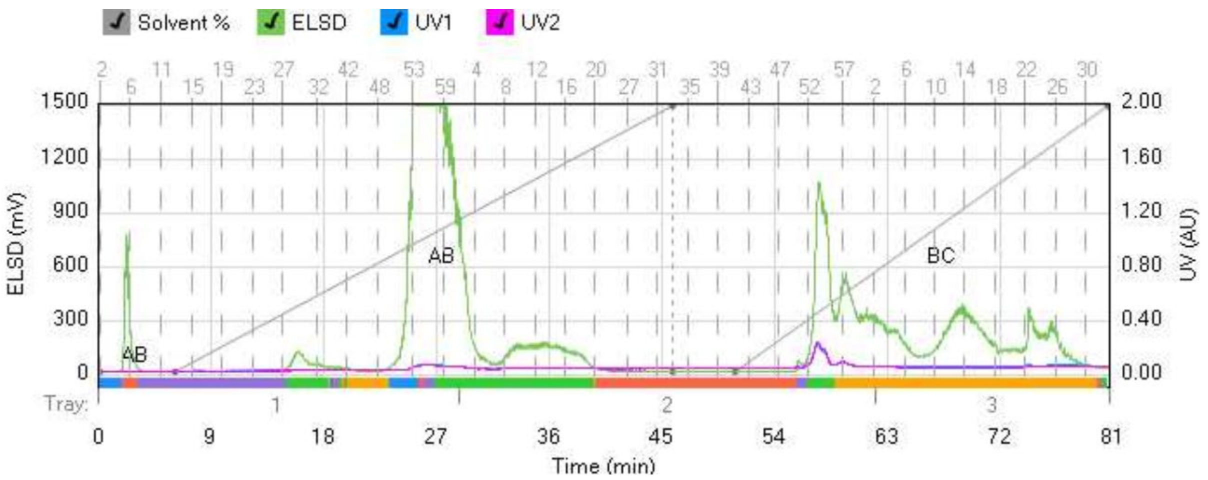

Figure S7. Flash chromatogram of methanol 90% fraction.

Table S1. First ten SMART 2.1 results for the major compound of mixture **M-6A.**

Name	Smiles	Source	Cosine score	MW
Quercetin	O=c1c(O)c(-c2ccc(O)c(O)c2)oc2cc(O)cc(O)c12	ACD_Labs	0.99869391946663 74	302.0
Quercetin Dihydrate	O.O=c1c(O)c(-c2ccc(O)c(O)c2)oc2cc(O)cc(O)c12	ACD_Labs	0.99869391946663 74	320.1
Quercetin	C1=CC(=C(C=C1C2=C(C(=O)C3=C(C=C(C=C3O2)O)O)O)O)O	ACD_Labs	0.99869391946663 74	302.0
"""MLS002153851-01!2-(3,4-dihydroxyphenyl)-3,5,7-trihydroxy-4H-chromen-4-one dihydrate117-39-5"""	Oc1cc(O)c2c(=O)c(O)c(oc2c1)c3ccc(O)c(O)c3.O	ACD_Labs	0.99869391946663 74	320.1
"ReSpect:PT104090 Quercetin|Quer|3,3',4',5,7-pentahydroxyflavone|Flavin meletin|Kvercetin|Meletin|Quercetol|Quercetine|Quertine|Quercitin|Sophoretin|Xanthaurine|3',4',5,7-Tetrahydroxyflavan-3-ol|2-(3,4-Dihydroxyphenyl)"	C1=CC(=C(C=C1C2=C(C(=O)C3=C(C=C(C=C3O2)O)O)O)O)O	ACD_Labs	0.99869391946663 74	302.0
"1,3,5,6-Tetrahydroxyxanthen-9-One"	O=c1c2ccc(O)c(O)c2oc2cc(O)cc(O)c12	ACD_Labs	0.97382986729509 11	260.0
"1,3,8-Trihydroxy-[1]Benzofuro[2,3-B]Chromen-11-One"	O=c1c2c(O)cc(O)cc2oc2oc3cc(O)ccc3c12	ACD_Labs	0.96898006475309 01	284.0
6-Hydroxyluteolin	O=c1cc(-c2ccc(O)c(O)c2)oc2cc(O)c(O)c(O)c12	ACD_Labs	0.95866857401569 94	302.0
Luteolin	O=c1cc(-c2ccc(O)c(O)c2)oc2cc(O)cc(O)c12	ACD_Labs	0.95600445326754 24	286.0
Luteolin	O=C1C2=C(C=C(C=C2OC(C3=CC=C(C(O)=C3)O)=C1)O)O	ACD_Labs	0.95600445326754 24	286.0

Table S2. First tenSMART 2.1 results for the major compound of mixture **M-6B.**

Name	Smiles	Source	Cosine score	MW
3-*O*-Methylquercetin	COc1c(-c2ccc(O)c(O)c2)oc2cc(O)cc(O)c2c1=O	ACD_Labs	0.97944410557808 37	316.1
3-*O*-methylquercetin	O=C1C2=C(O)C=C(O)C=C2OC(C3=CC(O)=C(O)C=C3)=C1OC	ACD_Labs	0.97944410557808 37	316.1
Hispidulin	COc1c(O)cc2oc(-c3ccc(O)cc3)cc(=O)c2c1O	ACD_Labs	0.94928334510070 88	300.1
NCGC00167728-05!5,7-dihydroxy-2-(4-hydroxyphenyl)-6-methoxychromen-4-one	COC1=C(O)C2=C(OC(=CC2=O)C3=CC=C(O)C=C3)C=C1O	ACD_Labs	0.94928334510070 88	300.1
4',7-Dihydroxy-3-Methoxyflavone	COc1c(-c2ccc(O)cc2)oc2cc(O)ccc2c1=O	ACD_Labs	0.94867195767113 18	284.1
4'-Hydroxywogonin	COc1c(O)cc(O)c2c(=O)cc(-c3ccc(O)cc3)oc12	ACD_Labs	0.94353334799565 42	300.1
2-(3,4-Dihydroxyphenyl)-5,6,7-Trihydroxy-3-Methoxychromen-4-One	COc1c(-c2ccc(O)c(O)c2)oc2cc(O)c(O)c(O)c2c1=O	ACD_Labs	0.94287655866651 86	332.1
3-Methoxy-7-Hydroxy-3',4'-Methylenedioxyflavone	COc1c(-c2ccc3c(c2)OCO3)oc2cc(O)ccc2c1=O	ACD_Labs	0.93662853344452 61	312.1
4'-methylgossypetin	COc1ccc(-c2oc3c(O)c(O)cc(O)c3c(=O)c2O)cc1O	Jeol	0.93661981783319 78	332.1
5-hydroxy-3,8-dimethoxy-3',4':6,7-bismethylenedioxyflavone	COc1c(-c2ccc3c(c2)OCO3)oc2c(OC)c3c(c(O)c2c1=O)OCO3	Jeol	0.93648437938095 12	386.1

Table S3. First tenSMART 2.1 results for the minor compound of mixture **M-6B.**

Name	Smiles	Source	Cosine score	MW
5,7,3',5'-Tetrahydroxy-3,4'-Dimethoxyflavone	COc1c(O)cc(-c2oc3cc(O)cc(O)c3c(=O)c2OC)cc1O	ACD_Labs	0.931111703	346.1
garcihombronone D	COc1cc2oc3c(OC)c(O)cc(O)c3c(=O)c2cc1O	Jeol	0.929443719	304.1
5-hydroxy-3,6,7-trimethoxy-2-(3,4,5-trimethoxyphenyl)-4H-chromen-4-one	O=C1C2=C(O)C(OC)=C(OC)C=C2OC(C3=CC(OC)=C(OC)C(OC)=C3)=C1OC	ACD_Labs	0.925304958	418.1
5,6-Dihydroxy-2-(4-Hydroxyphenyl)-7,8-Dimethoxychromen-4-One	COc1c(O)c(O)c2c(=O)cc(-c3ccc(O)cc3)oc2c1OC	ACD_Labs	0.925237879	330.1
4'-methylgossypetin	COc1ccc(-c2oc3c(O)c(O)cc(O)c3c(=O)c2O)cc1O	Jeol	0.924403668	332.1
1,6,7-Trihydroxy-2,3-Dimethoxyxanthone	COc1cc2oc3cc(O)c(O)cc3c(=O)c2c(O)c1OC	ACD_Labs	0.923919173	304.1
pyramidamycin B	COc1c(O)ccc(C(N)=O)c1O	ACD_Labs	0.923246482	183.1
pruniflorone O	COc1c(O)cc2oc3ccc(O)c(O)c3c(=O)c2c1OC	Jeol	0.922376422	304.1
Mearnsetin	COc1c(O)cc(-c2oc3cc(O)cc(O)c3c(=O)c2O)cc1O	ACD_Labs	0.915100135	332.1
2-(3,4-Dihydroxyphenyl)-5,6,7-Trihydroxy-3-Methoxychromen-4-One	COc1c(-c2ccc(O)c(O)c2)oc2cc(O)c(O)c(O)c2c1=O	ACD_Labs	0.911989886	332.1

Table S4. First ten SMART 2.1 results for the major compound of mixture **M-6C.**

Name	Smiles	Source	Cosine score	MW
3',5,7-Trihydroxy-3,4',5',6-Tetra-methoxyflavone	COc1cc(-c2oc3cc(O)c(OC)c(O)c3c(=O)c2OC)cc(O)c1OC	ACD_Labs	0.966581261	390.1
5,7-dihydroxy-2-(3-hydroxy-4,5-dimethoxyphenyl)-3,6-di-methoxy-4H-chromen-4-one	OC1=C(C(C(OC)=C(C2=CC(O)=C(OC)C(OC)=C2)O3)=O)C3=CC(O)=C1OC	ACD_Labs	0.966581261	390.1
CHEMBL3407503	COc1cc(-c2oc3cc(O)c(OC)c(O)c3c(=O)c2OC)cc(OC)c1OC	ACD_Labs	0.963569815	404.1
5,7-dihydroxy-3,6-dimethoxy-2-(3,4,5-trimethoxyphenyl)-4H-chromen-4-one	O=C1C2=C(O)C(OC)=C(O)C=C2OC(C3=CC(OC)=C(OC)C(OC)=C3)=C1OC	ACD_Labs	0.963569815	404.1
garcihombronone D	COc1cc2oc3c(OC)c(O)cc(O)c3c(=O)c2cc1O	Jeol	0.959102406	304.1
NCGC00385661-01!3,5,7,8-tetra-methoxy-2-(3,4,5-trimethoxy-phenyl)chromen-4-one	COC1=CC(=CC(OC)=C1OC)C2=C(OC)C(=O)C3=C(OC)C=C(OC)C(OC)=C3O2	ACD_Labs	0.953509138	432.1
Combretol	COc1cc(O)c2c(=O)c(OC)c(-c3cc(OC)c(OC)c(OC)c3)oc2c1	ACD_Labs	0.949210342	388.1
Brickellin	COc1cc(O)c(-c2oc3cc(OC)c(OC)c(O)c3c(=O)c2OC)cc1OC	ACD_Labs	0.947090565	404.1
5,7-Dihydroxy-3,3',4',6-Tetra-methoxyflavone	COc1ccc(-c2oc3cc(O)c(OC)c(O)c3c(=O)c2OC)cc1OC	ACD_Labs	0.94702507	374.1
2-(3,4-dimethoxyphenyl)-5,7-di-hydroxy-3,6-dimethoxy-4H-chromen-4-one	O=C1C2=C(O)C(OC)=C(O)C=C2OC(C3=CC=C(OC)C(OC)=C3)=C1OC	ACD_Labs	0.94702507	374.1

Table S5. First ten SMART 2.1 results for the minor compound of mixture **M-6C.**

Name	Smiles	Source	Cosine score	MW
5,7-dihydroxy-3',4',5'-trimethoxyflavone	COc1cc(-c2cc(=O)c3c(O)cc(O)cc3o2)cc(OC)c1OC	Jeol	0.980856037	344.1
Pmf	COc1cc(OC)c2c(=O)cc(-c3cc(OC)c(OC)c(OC)c3)oc2c1	ACD_Labs	0.969987497	372.1
Corymbosin	COc1cc(O)c2c(=O)cc(-c3cc(OC)c(OC)c(OC)c3)oc2c1	ACD_Labs	0.968526357	358.1
Chrysosplenol G	COc1cc(O)c2c(=O)c(OC)c(-c3cc(O)c(OC)cc3OC)oc2c1	ACD_Labs	0.963094821	374.1
Lethedocin	COc1cc(O)c2c(=O)cc(-c3cc(O)c(OC)c(OC)c3)oc2c1	ACD_Labs	0.961915005	344.1
Myricetin 3,3',4'-Trimethylether	COc1cc(-c2oc3cc(O)cc(O)c3c(=O)c2OC)cc(O)c1OC	ACD_Labs	0.946998833	360.1
5-hydroxy-2-(3-hydroxy-4,5-dimethoxyphenyl)-3,7-dimethoxy-4H-chromen-4-one	O=C1C2=C(O)C=C(OC)C=C2OC(C3=CC(O)=C(OC)C(OC)=C3)=C1OC	ACD_Labs	0.944809424	374.1
5-Hydroxy-2-(4-Hydroxy-3,5-Dimethoxyphenyl)-6,7-Dimethoxychromen-4-One	COc1cc(-c2cc(=O)c3c(O)c(OC)c(OC)cc3o2)cc(OC)c1O	ACD_Labs	0.943506297	374.1
"MLS000863593-01!5-hydroxy-2-(4-hydroxy-3,5-dimethoxyphenyl)-6,7-dimethoxychromen-4-one"	COc1cc(cc(OC)c1O)c2cc(=O)c3c(O)c(OC)c(OC)cc3o2	ACD_Labs	0.943506297	374.1
5,7-Dihydroxy-3',4',5'-Trimethoxyflavone	COc1cc(-c2cc(=O)c3c(O)cc(O)cc3o2)cc(OC)c1OC	ACD_Labs	0.943118601	344.1

References

1. Morreel, K.; Dima, O.; Kim, H.; Lu, F.; Niculaes, C.; Vanholme, R.; Dauwe, R.; Goeminne, G.; Inzé, D.; Messens, E.; et al. Mass Spectrometry-Based Sequencing of Lignin Oligomers. *Plant Physiol.* **2010**, *153*, 1464–1478, doi:10.1104/pp.110.156489.
2. Morreel, K.; Kim, H.; Lu, F.; Dima, O.; Akiyama, T.; Vanholme, R.; Niculaes, C.; Goeminne, G.; Inze, D.; Messens, E.; et al. Mass Spectrometry-Based Fragmentation as an Identification Tool in Lignomics. *Anal. Chem.* **2010**, *82*, 8095–8105, doi:10.1021/ac100968g.
